# Supplementary material for: Multiscale modelling of desquamation in the interfollicular epidermis
Source: PLoS Comput Biol. 2022 Aug 29;18(8):e1010368. doi: 10.1371/journal.pcbi.1010368 (PMC9462764; doi:10.1371/journal.pcbi.1010368)
Supplement: S1 Text — A. Parameter determination for subcellular model. Sourcing and fitting the parameters for the subcellular model. B. Analysis of the enzyme system. Extended results for the subcellular model. C. Distinct proliferative cell niches can be represented by a homogeneous population in the multiscale model. Multiscale model results for two proliferative populations with different cell cycle lengths. (PDF) [file pcbi.1010368.s001.pdf]

S1 Text: Multiscale modelling of desquamation in the interfollicular epidermis

A Parameter determination for subcellular model

This section details how the rate parameters and concentrations of reactants for the subcellular system: Eq (16) to (21) (main text), derived in Section Model: A subcellular system (main text), were calculated. The values determined here are used for the single cell results only (Main paper: Results: Realistic adhesion degradation rates in the subcellular model require and effective concentration of enzyme). Enzyme concentration is then modified to become an effective concentration of enzyme as described in the main paper and further detailed in Section B. Rate parameters are further adjusted in the multiscale model, as described in Model: The multiscale model (main paper), to account for the increased proliferation rate used in the multicellular model.

A.1 The pH gradient in the corneum

The local pH is the input for the ODE system for the cell, and depends on cell location. The pH gradient over the corneum was obtained from a graph in Ohman and Vahlquist [14] who collected data from human forearm, abdomen, and calf skin using sello-tape and cyanoacrylate resin stripping, shown as the point data in Fig A. We fit a curve to the forearm, abdomen, and calf data using the sello-tape stripping data. The function fit for the pH gradient is as follows:

$$\mathrm{pH} = f_{\mathrm{pH}}(\xi) = 6.8482 - 0.3765\,\xi - 5.1663\,\xi^2 + 3.1792\,\xi^3\,, \tag{S1}$$

where $\xi \in [0, 1]$ is the height of the cell above the base of the corneum as a proportion of the expected corneum thickness, shown as the solid black line in Fig A.

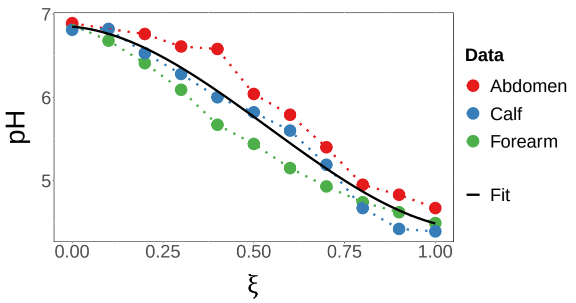

Fig A: The fit for the pH to the data from Ohman and Vahlquist [14].

A.2 Rate parameters

Eq (16) to (21) (main text) require five rate parameters: three for the interaction between the enzyme and the corneodesmosomes; k_{+1}, k_{-1}, and k_2, and two for the interaction between the enzyme and

LEKTI inhibitor; k_2 and k_{-3}. The data we use to estimate these parameters and their dependence on pH is given below.

KLK and corneodesmosome interaction

There are three rate parameters to determine for the KLK and corneodesmosome interaction given in Eq (14) (main paper). The first two, k_{+1} and k_{-1}, are the association and dissociation rates for the formation of the complex, C_S. The last rate parameter, k_2, is the rate at which the corneodesmosome complex is degraded.

The data available in the literature for this reaction is from Caubet et al. [4], who record the degradation of corneodesmosin and desmoglein 1, two proteins in corneodesmosomes, in neutral and acidic pH solution over a two hour period of incubation with KLK5. No LEKTI inhibitor was present in the Caubet et al. [4] experiments and each enzyme was tested separately, therefore our system of differential equations reduces to:

$$\frac{\mathrm{d}e}{\mathrm{d}t} = -k_{+1} s_0 e s + (k_{-1} + k_2)\, c_s \,, \tag{S2}$$

$$\frac{\mathrm{d}s}{\mathrm{d}t} = -k_{+1} e_T e s + k_{-1} \frac{e_T}{s_0} c_s \,, \tag{S3}$$

$$\frac{\mathrm{d}c_s}{\mathrm{d}t} = k_{+1} s_0 e s - (k_{-1} + k_2)\, c_s \,, \tag{S4}$$

$$\frac{\mathrm{d}p}{\mathrm{d}t} = k_2 \frac{e_T}{s_0} c_s \,. \tag{S5}$$

As the data has no information about c_s it is not possible to determine all rate parameters for this equation. Consequently, by making some assumptions about this interaction, we can instead determine a relationship between the parameters. In these experiments the KLK mass was 100 ng enzyme compared to 70 μg of the adhesive protein. Given the amount of adhesive protein is two orders of magnitude greater than the enzyme, we can assume that the rate of change in complex is negligible [11]. This is the quasi-steady state assumption from Briggs and Haldane [3]. Therefore we can assume the concentration of KLK5-CND compound is approximately constant, or:

$$\frac{\mathrm{d}c_s}{\mathrm{d}t} = 0, \tag{S6}$$

and, from Eq (S4), the amount of KLK and CND in compound is given by:

$$c_s = \frac{s_0}{K_M} e s, \tag{S7}$$

$$\text{where } K_M = \frac{k_{-1} + k_2}{k_{+1}}. \tag{S8}$$

We know the the total amount of free enzyme and enzyme in complex is conserved, so $e + c_s = 1$ (with no inhibition). Substituting Eq (S7) into this conservation equation, we get a formula for the amount of free enzyme, which we can then use to find an equation for c_s in terms of s:

$$e = \frac{1}{1 + \frac{s_0}{K_M} s}, \tag{S9}$$

$$c_s = \frac{s_0 s}{K_M + s_0 s}. \tag{S10}$$

Substituting these formulae for c_s and e into Eq (S3) produces the quasi-steady state equation for the degradation rate of the adhesive protein in terms of s:

$$\frac{\mathrm{d}s}{\mathrm{d}t} = -\frac{k_2 e_T s}{K_M + s_0 s}. \tag{S11}$$

Unit	CDSN	DSC1	KLK5
Weight in grams [μg]	70	70	0.1
Molecular weight [kDa]	54	100	34
Weight in moles [pmol]	1,300	700	3
Concentration [μM]	18,600	10,000	41

Table A: Conversion from weights to concentrations for the proteins and enzymes used in the Caubet et al. [4] paper. The experiments were run in volumes of 70 μL (email communication with M. Simon).

Parameter	Value
K_M [M]	4.60×10^{-5}
k_2 [hr^{-1}]	2.29×10^{3}
k_{+1} [M^{-1}.hr^{-1}]	4.97×10^{7}

Table B: Fitted parameter values for the interaction between enzyme and adhesion protein.

Solving this equation with initial condition $s(t=0)=1$, we get the following relationship between the proportion of remaining protein, s, and t:

$$K_M \log(s) + s_0 (s-1) = -k_2 e_T t\,. \tag{S12}$$

This equation can now be fitted to adhesion degradation data to determine K_M and k_2.

As mentioned above, Caubet et al. [4] determined the degradation over time of two adhesive proteins. The experiments used pH values of 5.6 and 7.2, however, as the authors point out, there appears to be little variation between the two in the data. Additionally, this data set is small and so it is not reliable to calculate fits with high confidence considering the different pH levels separately. Consequently, for this reaction we assume the effect of pH is negligible.

We can use the Caubet et al. [4] data to determine K_M and k_2 using Eq (S12). However, to maintain unit consistency with the rate parameter k_{+3} (Eq (S14)), it is necessary to first convert the weight to a Molarity. In order to convert concentrations to a molar quantity, we require the molecular weight of each protein and enzyme. Using molecular weights from Caubet et al. [4] we calculate the molar quantities shown in Table A. Concentrations are then determined by taking the molar weight and dividing by the volume for the experiment to give the values shown in Table A.

The calculated fits are given in Table B and shown, with the data, in Fig B1. Two lines can be seen on the plot, one for each adhesion protein (CDSN or DSC1), as each of the adhesion proteins has a different initial concentration (s_0), and consequently, from Eq (S12), the degradation curve will be different for each protein.

Given these values of K_M and k_2, we now need to determine the values of k_{+1} and k_{-1}. We have the following relationship between k_{+1} and k_{-1}, from Eq (S8):

$$k_{+1} = \frac{1}{K_M}(k_{-1} + k_2)\,. \tag{S13}$$

Given that K_M is on the order of 10^{-5} and k_2 is on the order of 10^3, this makes k_{+1} approximately five to eight orders of magnitude greater than k_{-1}. Consequently, we assume that the value of k_{-1} is negligible and approximate $k_{-1}=0$. Solving the full ODE system for different values of k_{-1} at both acidic and neutral pH supports this assumption. As can be seen in Fig B2, the variation in $s(T)$ is less than 0.01% for either pH. Consequently, we get the the value for k_{+1} shown in Table B.

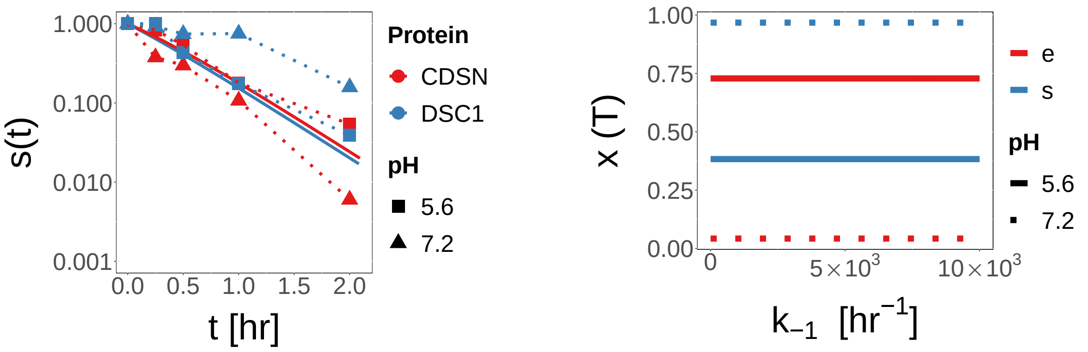

(1) Fit for K_M and k_2.

(2) Solution at $T = 50$ hours for varying k_{-1}

Fig B: (1) The fits to the adhesion degradation data. (2) The proportion of a and e, the amount of CND and free KLK5, after $T = 50$ hours for different values of k_{-1} showing that the variation with k_{-1} is negligible.

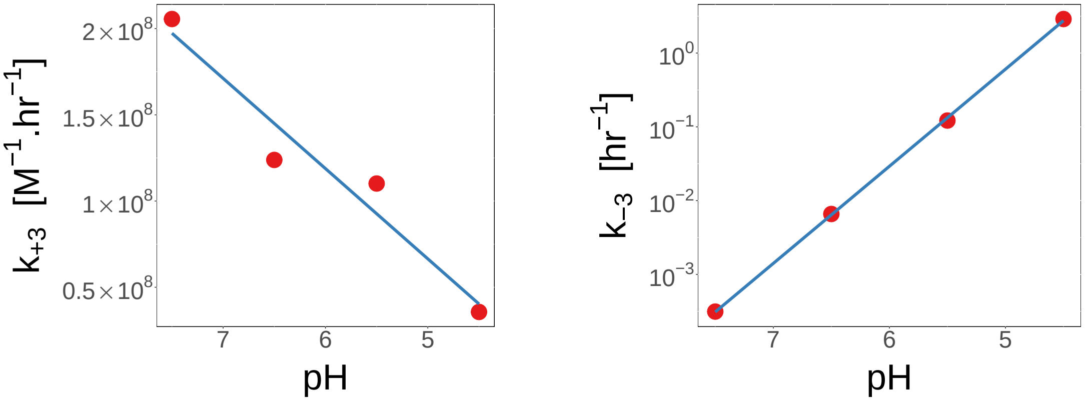

(1) Association rate parameter (k_{+3})

(2) Dissociation rate parameter (k_{-3})

Fig C: Fits to the association and dissociation data for the KLK5-LEKTI interaction (blue line). Data (red dots) is taken from Deraison et al. [6].

KLK5 and LEKTI interaction

Chemical Eq (15) (main paper) has two rate parameters: k_{+3} and k_{-3}, the association and dissociation rates for the binding between KLK5 and LEKTI respectively. A study by Deraison et al. [6] determined association and dissociation constants (k_{+3} and k_{-3}) at different pH from *in vitro* experiments. Fitting models to this data, shown in Fig C, produces the following equations for k_{+3} and k_{-3} as functions of pH:

$$k_{+3} = f_{+3}(\text{pH}) = (5.2\,\text{pH} - 19.5) \times 10^7\ [\text{M}^{-1}.\text{hr}^{-1}], \tag{S14}$$

$$k_{-3} = f_{-3}(\text{pH}) = 2.3 \times 10^6\, \text{e}^{-3.0\text{pH}}\ [\text{hr}^{-1}]. \tag{S15}$$

A.3 Concentrations of KLK, LEKTI, and CND

There is limited *in vivo* data for the concentrations of KLK, LEKTI, and CND in the corneum. The available data and estimates we make for each reactant are detailed below.

KLK enzyme

Several studies have investigated the weight of KLK in epidermal tissue. The data is provided as a weight of free enzyme per weight of dry corneum tissue. Consequently, it does not account for enzyme in complex and provides no spatial component to the concentrations. It is necessary, for the purposes of the model, to convert these amounts to molar concentrations in extracellular space.

Komatsu et al. [12] measured levels of different KLK enzymes for healthy skin and skin with Netherton syndrome (NS). In healthy skin, the study detected a total enzyme amount of 30 ng.mg^{-1} dry weight. Of this, 19.1 ng.mg^{-1} was trypsin-like KLKs, 3.1 ng.mg^{-1} was KLK5. In order to convert this to a molar concentration we need two more pieces of information: the molecular weight of KLK5, and the volume of extracellular water per mg dry weight of stratum corneum tissue. The molecular weight of active KLK5 is 33 kDa [8], making 3.1 ng.mg^{-1} of KLK5 equivalent to 93.9 fmol.mg^{-1}. We can approximate the volume of extracellular water to dry tissue weight using the water content of the tissue and the volume ratio of cell to extracellular space in the corneum.

The water content of the corneum varies between deep and superficial tissue, ranging from 65–70% in the granular layers (and below) to 30–40% at the surface of the corneum [7] Consequently, we estimate it at 50%, or 0.5 g.g^{-1}, making the water weight equal to the dry tissue weight. Given the density of water is 1 mL.g^{-1}, the volume of water in millilitres is equal to the weight of dry tissue in grams. However, this water content includes both the intracellular and extracellular water. In order to account for this, we estimate the ratio of intracellular to extracellular water by the volume ratio of the cell to extracellular space. We know the height of a corneocyte is around 300 nM [2], and it has been determined that the extracellular space between the cell sheets in the upper corneum is 44 nM [1]. Consequently, we estimate the volume of extracellular space, and hence extracellular water, to be approximately 13% of the total volume. Therefore, our conversion from dry tissue weight to water volume in mL is 0.13 times the dry tissue weight in grams, making 93.9 fmol.mg^{-1} KLK5 in dry tissue roughly equivalent to an enzyme concentration of 0.723 μM.

A summary of these values is given in the main paper in Table 2.

LEKTI inhibitor

LEKTI is synthesised by the cell as a large protein, which is then broken down into smaller fragments before release into the extracellular space [8]. Particular LEKTI fragments are better at inhibiting particular KLK enzymes. Fortugno et al. [8] measured inhibition effectiveness of different LEKTI fragments for different KLK enzymes, as well as their molar quantities in human epidermis. The key result for the purposes of this study was that the LEKTI fragments most effective at inhibiting KLK5 were present in the same molar quantities as KLK5. Consequently, we set $i_T = e_T$ in normal epidermis.

Corneodesmosomes

The *in vivo* data on corneodesmosomes provides counts of associated proteins across the edges of cells. Igawa et al. [9] counted 16 proteins per μm on the peripherals of cells and 10 proteins per μm in the central regions of cells. In order to convert this into a concentration we need to determine the number of proteins per unit of extracellular volume. We approximate a cell as a rectangle of 30 μm width and 0.3 μm height [2] and use the definition of peripheral and central from Igawa et al. [9] where the peripheral regions extend a quarter of the way into the cell. As above, we know the extracellular space is 0.044 μm [1]. From this, we can estimate the concentration of proteins in the space between two stacked cells to be 1.6×10^5 proteins in 39.6 μm^3. This is equivalent to 4.0×10^{18} proteins.L^{-1}, or 6.6 μM. This value is a very rough estimation, but provides an idea of the order of magnitude of the protein concentration. We note this is only one order of magnitude greater than the enzyme concentration. A summary of these values is given in the main paper in Table 3.

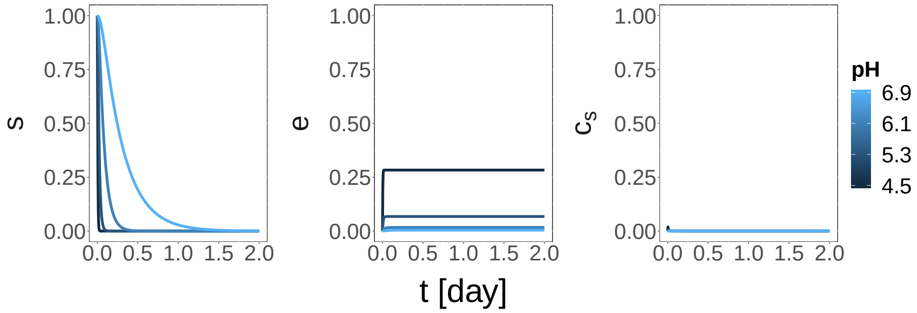

Fig D: Results for the system at varying pH. Reactant concentrations are $e_T = i_T = 0.723$ μM and $s_0 = 6.6$ μM.

B Analysis of the enzyme system

In this supplementary section we provide further analysis of the single cell dynamics of the ODE system, external to the multicellular model. We consider the system response at different pH, and to different reactant concentrations assuming a specified upwards velocity of the cell, determined using cell migration times and stratum corneum thickness data.

This is intended as a supplement to the results given in the main paper (Results: Realistic adhesion degradation rates in the subcellular model require an effective concentration of enzyme). Specifically, these supplementary results provide more detail behind the use of an effective concentration of enzyme.

B.1 Decreasing pH increases degradation of adhesion but does not match expected degradation rates

The first result we look at is the degradation of the substrate at varying levels of pH. These results are shown in Fig D. The first observation is the increased rate of degradation with decreasing pH. This is in support of the hypothesised system, as the high pH represents the activity at deep epidermis while low pH is the equivalent of superficial epidermis. This is not a surprising result, as the rate parameters for the inhibition reaction should free more enzyme from complex at lower pH. In Fig C, at high pH (deep epidermis), the ratio of association to dissociation of inhibitor complex is on the order of magnitude of 10^{11}, while at low pH, this ratio on the order of 10^{7}. Consequently, we expect much more inhibitor complex at high pH, decreasing the amount of enzyme available to interact with the CND.

A second observation from Fig D is the rate of degradation of the corneodesmosomes (s). We know the migration time of a cell through the stratum corneum is around 20 days, and so would expect the degradation to occur at a similar time scale. The results show much faster time scales, closer to a day. We explore this in more detail below, by considering the degradation of a cell migrating through the pH gradient.

B.2 Solutions for a migrating cell highlight the effect of limited diffusion

In order to better understand this system, we can solve it for a cell migrating through the pH gradient. This provides a more realistic representation of the degradation of the adhesion proteins. From Roberts and Marks [15], we know the migration of the cell through the corneum takes around 18.5–26.5 days in human epidermis. We assume that the cell velocity is approximately constant through the stratum corneum, and estimate the migration time as 20 days. This gives us an approximate velocity, normalised by stratum corneum thickness, of $v_\xi = 0.05$ day^{-1}.

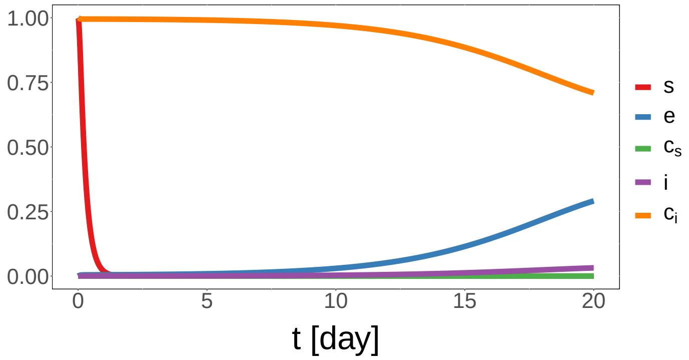

Fig E: The solutions for a cell migrating through the pH gradient.

Fig E shows the results for a cell migrating through the pH gradient over the expected 20 days migration time. As we saw in Fig D, the substrate degrades too fast to see the effect of the pH gradient.

If we consider the spatial component to degradation—the degradation of corneodesmosomes occurs initially on the horizontal surfaces of the cells—we can determine if the data matches the degradation time of these planar proteins. Igawa et al. [10] found that the proteins on the horizontal surface were essentially degraded at the third layer. If we assume that the stratum corneum depth is 10–20 layers, we would therefore expect these proteins to be degraded around days 3–6. This expected degradation is still slower than the results seen in the model, which indicates that either the rate parameters are wrong (k_{+1} and k_2), or that the mechanism is wrong.

We know there is a parameter regime in which the system produces the desired results, as can be seen in Fig F. We also know more processes occur in the system than this one interaction, which likely affect the reaction. Igawa et al. [9] hypothesise that tight junctions at the peripheral of the cells act as a barrier to the enzyme-corneodesmosome interaction at peripheral sites. Our results indicate that, assuming rate parameters are at the right order of magnitude, this could have a significant effect on the degradation rate. Additionally, similar effects may even be occurring at the central regions of the cell, with the diffusion of the enzyme potentially limited by the intact corneodesmosomes themselves or the lipids also residing in the extracellular space. By limiting the diffusion of the enzyme, the system is no longer mixed and consequently the mass action model does not hold. In order to compensate for this limited diffusion effect, without incorporating new processes in the model, we instead propose the use of an *effective concentration of enzyme*. This will be explored in the next section.

B.3 Incorporating an effective concentration of enzyme reproduces observed desquamation rates

We investigate changing the concentrations of the enzyme, and subsequently the inhibitor, or the substrate. We consider different orders of magnitude of the reactants, rounding our base concentrations: $s_0 = 10$ μM and $e_T = i_T = 1$ μM. The results for varying s_0 and e_T can be seen in Fig F.

The first reactant we vary is the amount of KLK enzyme, or e_T. By decreasing e_T, we are effectively limiting the amount of enzyme available for degradation of corneodesmosomes, and consequently this is a way to simulate limited diffusion. As we vary e_T we also maintain $i_T = e_T$, as we want sufficient LEKTI to bind with the KLK.

From Eq (17) (main paper), we can see that decreasing e_T directly affects the effective rate parameter for the degradation of s, which is given by $k_{+1}e_T$. However, it also will indirectly affect the amount of free enzyme. The equivalent decrease in i_T can significantly decrease the upper bound on i (given by i_T/s_0), and consequently could be expected to indirectly decrease the rate of the formation of inhibitor complex, and increase e, as given by Eq (16) (main paper).

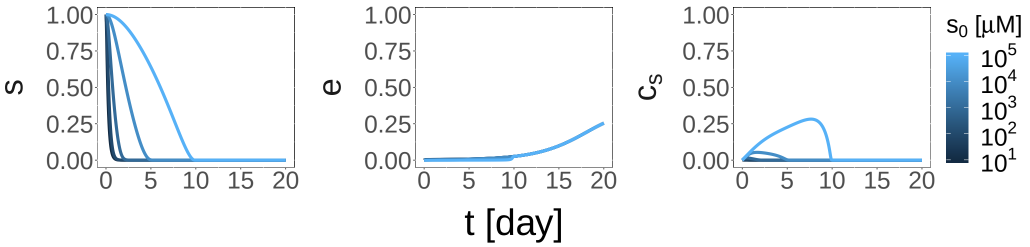

(1) Varying s_0 ($i_T = e_T = 1$ μM).

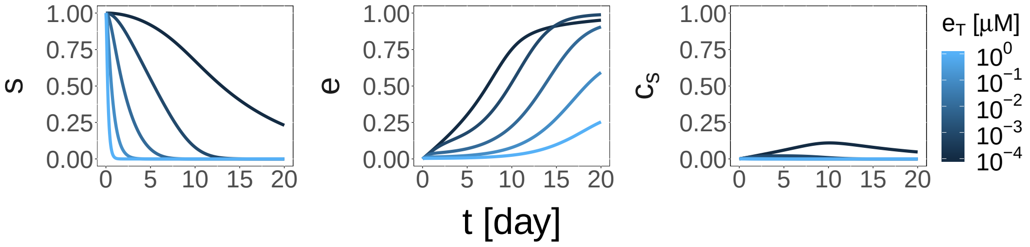

(2) Varying e_T ($i_T = e_T$ and $s_0 = 10$ μM).

Fig F: The effect of varying concentrations on the system dynamics and rate of degradation of adhesion.

	**Results**				
**Quantity**	$e_T = 1.0$	$e_T = 10^{-1}$	$e_T = 10^{-2}$	$e_T = 10^{-3}$	$e_T = 10^{-4}$
$T_{s=0.25}$ [days]	0.4	1.1	3.2	7.8	19.3

Table C: Quantifying the effect of degradation of adhesion with increasing e_T (and i_T). The units of e_T are μM, and $i_T = e_T$. Results are the time taken for the substrate (adhesion complex) to get below 25% of its initial concentration.

The results for decreasing e_T can be seen in Fig F2. As would be expected, decreasing the total amount of enzyme in the system increases the rate of degradation of the substrate. Also seen in Fig F2, increasing e_T decreases the value of e. This is due to the indirect effect on e from the increasing upper bound on i, as mentioned above. However, this does not have as large an effect on the degradation of s as the increased effective rate parameter $(k_{+1}e_T)$ with increased e_T.

We can better quantify the change in degradation by considering the point at which the substrate drops below 25%, or $s \leq 0.25$. These values are shown in Table C. As can be seen in the table, by scaling the enzyme by a factor of 10^{-4} we can reproduce the expected rate of degradation of adhesion.

Next we consider the effect of increasing the amount of corneodesmosome, or s. This corresponds to varying the amount of adhesion between cells. Unlike decreasing e_T, it does not directly relate to a limited diffusion mechanism, but does affect rate parameters.

Increasing s_0 increases the effective rate parameters for formation of the complexes, which are given by $k_{+1}s_0$ and $k_{+3}s_0$ in Eq (16) (main paper). This would appear to decrease the amount of free enzyme available to degrade the complex. However, similarly to the effect of increasing i_T mentioned above, an increase in s_0 can significantly decrease the upper bound on i (given by i_T/s_0) and cause an increase in e, reversing the effect on the change of rate for the formation of inhibitor complex. Physically this makes sense, as changing the initial substrate concentration should not affect the rate of enzyme formation of complex with the inhibitor, just the amount of free enzyme.

The results for increasing s_0 are shown in Fig F1. We see that, while there is substrate remaining and available for complex, essentially all the enzyme is in complex with either the substrate or the

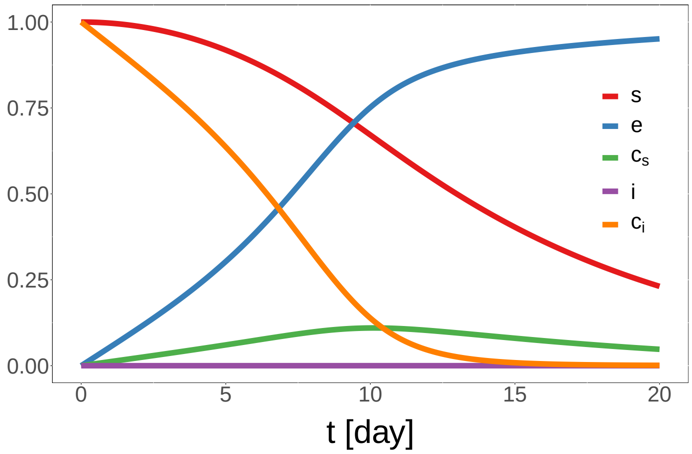

Fig G: Model results incorporating an effective concentration of enzyme ($e_T = 0.1$ nM).

	Results				
**Quantity**	$i_T = e_T$	$i_T = 0.75e_T$	$i_T = 0.5e_T$	$i_T = 0.25e_T$	$i_T = 0.0e_T$
$T_{s=0.25}$ [days]	19.3	17.9	16.3	14.8	13.0
$\int_0^{20} e\ dt$	12.3	13.7	15.1	16.7	18.4

Table D: Quantification of the results for abnormal versus normal system. The units of i_T are μN and $e_T = 0.1$ nM. Integrals are taken for the single cell across the 20 days.

inhibitor, as expected. The main conclusion to draw from Fig F1 is that we are unable to prolong the degradation of the adhesion to 20 days; even with an increase in s by a factor of 10^4, full degradation occurs in half the time we expect it to.

Fig F shows a reduced concentration of e_T is able to reproduce the rates expected for the desquamation process. Additionally, the reduction of this parameter is a computationally efficient way to simulate the effect of limited diffusion, which would also reduce the available enzyme to bind to the substrate. In order to produce the expected results, we use an order of magnitude of $e_T = 0.1$ nM in the main paper. An example of a full solution of the system with $e_T = 0.1$ nM, $i_T = e_T$, and $s_0 = 10$ μM is shown in Fig G.

B.4 Modelling disease

We can use this model to investigate the changed dynamics due to *Netherton Syndrome (NS)*, a disorder that mutates the gene for the LEKTI inhibitor, on a single cell. An analysis of the disease for the multiscale model is given in the main paper. Presumably as a result of the reduced amount of inhibitor, KLK levels are elevated in NS patients. Komatsu et al. [12] recorded KLK levels in the NS patients between 157% to 486% that of healthy stratum corneum.

We can consider the effect of reduced LEKTI inhibitor on the model by reducing the concentration, i_T. These results can be seen in Fig H. As would be expected, decreasing the amount of inhibitor decreases the amount of enzyme in complex with the inhibitor, and consequently increases the degradation rate of the substrate. We can again quantify these results by comparing the time at which the substrate drops below 25%, and by determining the amount of free enzyme over the whole stratum corneum for comparison to the data from Komatsu et al. [12]. These values are shown in Table D.

As can be seen in Table D, the worst case scenario would produce stratum corneum with a reduced

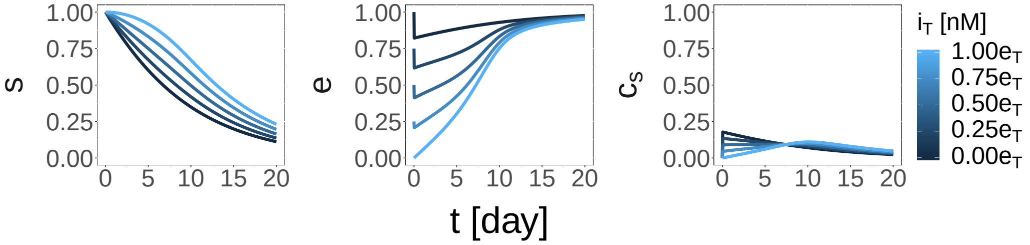

Fig H: Varying i_T ($e_T = 0.1$ nM, $s_0 = 10$ μM).

depth of 33% and 50% more free KLK. This is the smallest amount of extra KLK observed in NS patients in the data from Komatsu et al. [12], and therefore is likely underpredicting the effect of the reduced LEKTI. However, if we consider the results in Fig F2, we can see that the proportion of free enzyme at lower total e_T concentrations is much higher than at high e_T concentrations. Consequently, if we were instead to compare the same integral ($\int_0^{20} e \, \mathrm{d}t$) with the original estimate of $e_T = 1$ μM, we get 1.2 for $i_T = e_T$ compared to 5.4 for $i_T = 0.75e_T$, which is an increase of 350% and much closer to the results seen experimentally. However, it is not possible to analyse the reduced depth at these concentrations as the substrate degrades too fast to produce these results.

These results tell us that, using the experimental values of enzyme concentration, it only requires small reductions in the amount of inhibitor (around 25%) to reproduce the higher weights of enzymes seen in NS stratum corneum experimentally. However, using the effective concentration of enzyme, we reduce the impact of the reduction of inhibitor.

C Distinct proliferative cell niches can be represented by a homogeneous population in the multiscale model

Experiments have shown evidence of two populations of proliferative cells in the basal layer with different cell cycle lengths [5, 13, 16]. We investigate the effect of two proliferative populations in our multiscale model by comparing different combinations of fast and slow cycling stem cell populations with the same harmonic mean: $H(\mathrm{T_C}) = 15$ hours. The harmonic mean ensures the overall proliferation rate is the same across each setup, as it accounts for the fact that fast cycling cells undergo more cycles than slow cycling cells. Due to this behaviour, we would expect an increased number of divisions to occur than would be inferred from the arithmetic mean. We also ran simulations using the same arithmetic mean (results not shown) which confirmed this.

To implement the two populations, we set 50% of the cells as fast cycling: $\mathrm{T_C} = \mathrm{T_1}$, and 50% as slow cycling: $\mathrm{T_C} = \mathrm{T_1} + \Delta\mathrm{T_C}$. We choose a range of values for $\Delta\mathrm{T_C}$ and determine $\mathrm{T_1}$ using the following equation:

$$\mathrm{T_1} = \frac{H_\mu - \Delta\mathrm{T_C}}{2} + \frac{\sqrt{\Delta\mathrm{T_C}^2 + H_\mu^2}}{2}, \tag{S16}$$

where $H_\mu = 15$ hours is the desired value of the harmonic mean. The allocation of the fast or slow cycle length to the stem cells was random. The cell lineage used is asymmetric division with one stem cell population; i.e. one stem cell produces one stem and one differentiated cell, making all stem cells in the system immortal. Consequently, the ratio of fast to slow cycling cells in the simulations persist at 50:50.

As can be seen in Fig I, increasing the cycle difference but maintaining the same harmonic mean produces the same steady state corneum thickness (τ_{ss}) and mean z velocities. This is because the harmonic mean accounts for the increased count of events occurring for cells with higher rates. If we calculate the average proliferation rate in each simulation it is found to be 1.60 divisions per day for

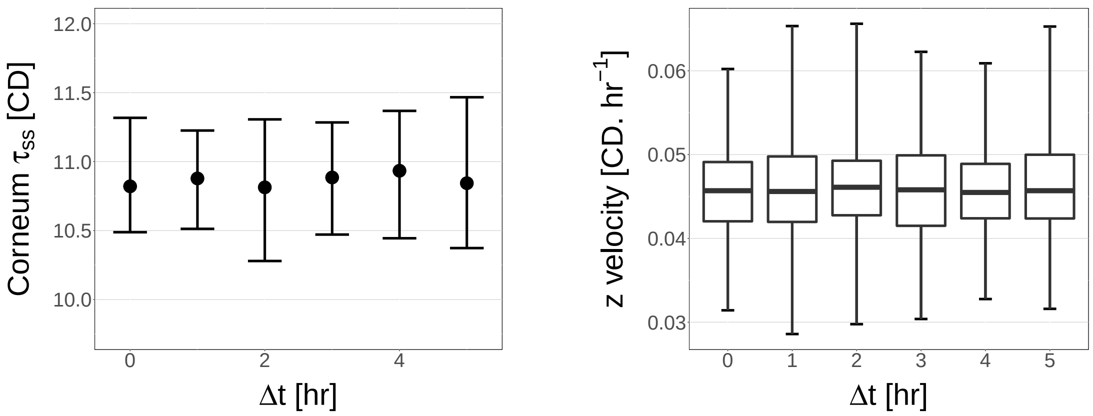

(1) (2)

Fig I: Results for simulations with two proliferative populations (two different cell cycle times). Δt is the difference in cycle time between the two populations, and the harmonic mean is the same for all configurations. (1) The steady state height of the corneum, τ_{ss}. The points show the mean and the bars indicate the minimum and maximums across the ten realisations of each setup. Note that the mean is approximately the same regardless of the difference in cycle time between the two populations as they have the same harmonic mean. (2) The cell velocities (excluding stem and top cells). The box plot shows the quartiles, and the error bars give the minimum and maximum across all cells and simulations. Again, this is approximately the same regardless of cell cycle difference due to each configuration having the same harmonic mean.

each setup. Consequently, it is logical that the average velocity remains the same and, given we are not modifying any aspect of desquamation, therefore the thickness is also the same. Given this result, we know we can approximate the expected steady state thickness of a system with two populations of proliferative cells by a single population system with cycle time equal to the harmonic mean of the two populations.

References

[1] A. Al-Amoudi, J. Dubochet, and L. Norlén. "Nanostructure of the Epidermal Extracellular Space as Observed by Cryo-Electron Microscopy of Vitreous Sections of Human Skin". In: *Journal of Investigative Dermatology* 124.4 (Apr. 2005), pp. 764–777. ISSN: 0022-202X. DOI: 10.1111/j.0022-202X.2005.23630.x.

[2] J. A. Bouwstra, A. de Graaff, G. S. Gooris, et al. "Water Distribution and Related Morphology in Human Stratum Corneum at Different Hydration Levels". In: *Journal of Investigative Dermatology* 120.5 (May 2003), pp. 750–758. ISSN: 0022-202X. DOI: 10.1046/j.1523-1747.2003.12128.x.

[3] G. E. Briggs and J. B. S. Haldane. "A Note on the Kinetics of Enzyme Action". en. In: *Biochemical Journal* 19.2 (1925), pp. 338–339. ISSN: 0264-6021, 1470-8728. DOI: 10.1042/bj0190338.

[4] C. Caubet, N. Jonca, M. Brattsand, et al. "Degradation of Corneodesmosome Proteins by Two Serine Proteases of the Kallikrein Family, SCTE/KLK5/hK5 and SCCE/KLK7/hK7". In: *Journal of Investigative Dermatology* 122.5 (May 2004), pp. 1235–1244. ISSN: 0022-202X. DOI: 10.1111/j.0022-202X.2004.22512.x.

[5] E. Clayton, D. P. Doupé, A. M. Klein, et al. "A Single Type of Progenitor Cell Maintains Normal Epidermis". en. In: *Nature* 446.7132 (Mar. 2007), pp. 185–189. ISSN: 0028-0836. DOI: 10.1038/nature05574.

[6] C. Deraison, C. Bonnart, F. Lopez, et al. "LEKTI Fragments Specifically Inhibit KLK5, KLK7, and KLK14 and Control Desquamation through a pH-Dependent Interaction". In: *Molecular Biology of the Cell* 18.9 (June 2007), pp. 3607–3619. ISSN: 1059-1524. DOI: 10.1091/mbc.e07-02-0124.

[7] M. Egawa, T. Hirao, and M. Takahashi. "In Vivo Estimation of Stratum Corneum Thickness from Water Concentration Profiles Obtained with Raman Spectroscopy". en. In: *Acta Dermato-Venereologica* 87.1 (2007), pp. 4–8. ISSN: 0001-5555. DOI: 10.2340/00015555-0183.

[8] P. Fortugno, A. Bresciani, C. Paolini, et al. "Proteolytic Activation Cascade of the Netherton Syndrome–Defective Protein, LEKTI, in the Epidermis: Implications for Skin Homeostasis". In: *Journal of Investigative Dermatology* 131.11 (Nov. 2011), pp. 2223–2232. ISSN: 0022-202X. DOI: 10.1038/jid.2011.174.

[9] S. Igawa, M. Kishibe, M. Murakami, et al. "Tight Junctions in the Stratum Corneum Explain Spatial Differences in Corneodesmosome Degradation". en. In: *Experimental Dermatology* 20.1 (Jan. 2011), pp. 53–57. ISSN: 1600-0625. DOI: 10.1111/j.1600-0625.2010.01170.x.

[10] S. Igawa, M. Kishibe, M. Murakami, et al. "Tight Junctions in the Stratum Corneum Explain Spatial Differences in Corneodesmosome Degradation". en. In: *Experimental Dermatology* 20.1 (2011), pp. 53–57. ISSN: 1600-0625. DOI: 10.1111/j.1600-0625.2010.01170.x.

[11] J. Keener and J. Sneyd. *Mathematical Physiology. I: Cellular Physiology.* eng. 2nd ed. Vol. 8/1. Interdisciplinary Applied Mathematics. New York, NY: Springer-Verlag New York, 2009. ISBN: 978-0-387-75846-6. DOI: 10.1007/978-0-387-75847-3.

[12] N. Komatsu, K. Saijoh, A. Jayakumar, et al. "Correlation between SPINK5 Gene Mutations and Clinical Manifestations in Netherton Syndrome Patients". In: *Journal of Investigative Dermatology* 128.5 (May 2008), pp. 1148–1159. ISSN: 0022-202X. DOI: 10.1038/sj.jid.5701153.

[13] G. Mascré, S. Dekoninck, B. Drogat, et al. "Distinct Contribution of Stem and Progenitor Cells to Epidermal Maintenance". In: *Nature* 489.7415 (Sept. 2012), pp. 257–262. ISSN: 1476-4687. DOI: 10.1038/nature11393.

[14] H. Ohman and A. Vahlquist. "In Vivo Studies Concerning a pH Gradient in Human Stratum Corneum and Upper Epidermis". In: *Acta Dermato-Venereologica* 74 (1994), pp. 375–379. DOI: 10.2340/0001555574375379.

[15] D. Roberts and R. Marks. "The Determination of Regional and Age Variations in the Rate of Desquamation: A Comparison of Four Techniques". In: *Journal of Investigative Dermatology* 74.1 (Jan. 1980), pp. 13–16. ISSN: 0022-202X. DOI: 10.1111/1523-1747.ep12514568.

[16] A. Sada, F. Jacob, E. Leung, et al. "Defining the Cellular Lineage Hierarchy in the Interfollicular Epidermis of Adult Skin". en. In: *Nature Cell Biology* 18.6 (June 2016), pp. 619–631. ISSN: 1465-7392. DOI: 10.1038/ncb3359.
