# Supplementary material for: Multiscale modelling of desquamation in the interfollicular epidermis
Source: PLoS Comput Biol. 2022 Aug 29;18(8):e1010368. doi: 10.1371/journal.pcbi.1010368 (PMC9462764; doi:10.1371/journal.pcbi.1010368)
Supplement: Attachment — Submitted filename: PLOS_Compbio_Oct21_commentstoauthors.pdf [file pcbi.1010368.s005.pdf]

Manuscript Review - Multiscale modelling of desquamation in the interfollicular epidermis

This manuscript describes the implementation and results of a multiscale model incorporating components representing i) the dynamics of corneodesmosome adhesion proteins between epidermal cells and ii) a multicellular overlappign sphere model where the adhesion forces between cells are informed by the protein substrate values calculated in part i). The model is used to examine the process of desquamation and its role in homeostasis and also, the effect of LEKT1 which can disrupt this process and is associated with skin disease.

General comments

The manuscript is thorough in its description of the two model components and is clearly written and presented. I am not a mathematician, but the equations and methodology underlying the models appears to be rigoruos and well justified. The author's have gone to considerable lengths to inform their model parameters from experimental data extracted from the literature.

Specific comments

Although the components of the multiscale model are individually well described, there is no explicit mntion of the time steps associated with the subcellular signalling model and the cell-based model. Some discussion of the native time scales associated with each component and the justification of the time splitting technique implemented should be provided (e.g. see Bhattacharya, P., Li, Q., Lacroix, D., Kadirkamanathan, V., & Viceconti, M. (2021). A systematic approach to the scale separation problem in the development of multiscale models. *PloS one*, *16*(5), e0251297 for a recent overview, though earlier related publications do exist).

Page 5 "*This model uses cell height to determine the local pH….*" This is confusing as "cell height" could refer to the physical dimension of a cell, rather than the co-ordinates in the z direction, so would suggest revising for clarity.

Page 5 "*We assume that the cell velocity is approximately constant through the stratum corneum….*" – confusing as it is later stated that cell movement is an emergent outcome of the multicellular model – or is this value assumed just for a preliminary calculation?

Page 5 – "*In particular, it has been hypothesised that tight junctions at the peripheral of cells…*" should read "peripherary?

Figure 3 needs a colour key/legend

Figure 8b – the difference between the two blue and red lines is unclear.

Regarding the results relating to *Distinct proliferative cell niches can be represented by a homogenous population*

Here is seems that the authours have compared simulations where i) cells are given the mean cycling time and ii) a subset of cells (representing stem cells) are randomly assigned a longer cycling time, and concluded that there is no difference. However, my understanding is that "stem cell niches" referred to regions where stem cells were spatially localised/grouped rather than randomly distributed over the cell membrane. In this regard, the authors have not actually explored the effect of spatially localised stem cell niches, and this should be made clear.

Regarding the results relating to *Heterogenous full recovery is more effective than homogenous partial recovery*
Here the authors explore a hypothetical treatment where the the intrinsic value of inhibitor in either all, or a subset of cells is altered, and this property is passed from mother to daughter cells at division. I am not aware how this would relate to a real treatment modality (where I would assume that there is some diffusion of drug from the deeper tissue, in the case of systemic delivery, or the surface in terms of a topical treatment). It would be desirable to see a little more discussion on how this particular computational exploration relates to real treatment for Netherton Syndrome (or possibly other skin disorders).

It is mentioned that only 10 simulations per case were carried out. Was this due to prohibitive computational resources requirements/run times? It would be intersting to see more information on this, even if only in Supplementary material.

There is no parameter sensitviity analysis provided on which to judge the robustness of the results. This should at least be discussed as a limitation to the work.

Model Description
In the subcellular model, it does not appear that new protein production, or degradation is accounted for. This may be justified by the timescales, but given that they are not explicitly stated, it is difficult to tell.

Page 19 paragraph 1 - typo – "…*which is discussed in the The multiscale model section*."

Another assumption is that extracellular space between cells does not vary with z position within the epidermis. This is unlikely to be the case, so should be mentioned as a simplication.

Page 21 paragraph 1 typo – "..*assume that any enzyi=me that is <u>not complex with</u> the inhibitor*…*

Page 22 – equation 19 – the formulation of the force terms should be justified/referenced.
