# Supplementary material for: Multiscale modelling of desquamation in the interfollicular epidermis
Source: PLoS Comput Biol. 2022 Aug 29;18(8):e1010368. doi: 10.1371/journal.pcbi.1010368 (PMC9462764; doi:10.1371/journal.pcbi.1010368)
Supplement: Attachment — Submitted filename: Miller2021PCB.pdf [file pcbi.1010368.s006.pdf]

In this paper Miller et al. present a computational model of epidermal tissue. The model is used to investigate how the thickness of the stratum corneum can be related to subcellular regulation of adhesion. The model is applied to a condition known as Netherton syndrome, a condition in which the removal of cells from the top-most layers is affected.

1. The introduction can better critique the state-of-the-art in the field so that the novelty and importance of the proposed model can be assessed.
2. Can the authors better identify what the aims of the work and try to focus the emphasis of the results towards addressing particular aims.
3. Details of the model equations need to provided in the main text.
4. Some of the simplifications made to make the model more efficient to simulate seem rather ad hoc. How do the authors know that the simplification are not affecting results in unanticipated ways? Is it worth simulating the model in 3D if one has to make such compromises? Are such simplifications made in the other models?

General

1. L2 Are all experimental data taken from human studies?
2. L103 Can the authors expand on why it is necessary to model subcellular processes controlling adhesion degradation? What scientific question is addressed by doing this? Perhaps this point can be addressed in the discussion.
3. L 111-127 - I did not find this overview helpful and suggest removing.
4. L131 - present the model equations. One of key proposed novelties of the paper is a model for desquamification. In L148 the rate parameters k_1 and k_2 are discussed but these have not been introduced in the main text.
5. L141 - is the constant velocity assumption valid (check by comparing with simulation results). I would expect to see a velocity gradient along the z axis.
6. L147 - expand on the results here.
7. L137 - the authors could outline the main equations in the results section.
8. L172 - by what factor is e_T reduced. This is not mentioned in the figure caption and not obvious in the appendix. Given the uncertainties in parameter estimates is it necessary to go into such detail about the parameter fitting here. This section detracts from the flow of the paper. Perhaps use a lower value and note this is the case. Then in the discussion go into some more detail, highlighting potential mechanisms that could results in an effective parameter.
9. L174 - provide a brief introduction to the multicellular model here (e.g. equations of motion, boundary conditions, cell cycle rules provide a table with parameter values in the main text etc). These details are crucial for the interpretation of the main results. Special focus ought

to be paid to how the molecular model couples to the sadhesion term in the multicellular model.

10. L191 Critique comparison with ref. [131] in the discussion section.
11. L194 - remove healthy!
12. L201 - what system?
13. L211-L225 the description of the results in the text is overly numerical. What is the significance of the described numbers? This section could be reduced, with the focus being on the key principles (emergence of homeostasis and dependency on key parameters).
14. L259 - this section is drifting somewhat from the main focus of the paper and seems somewhat incidental. Could the authors set the model up with a single proliferating cell population and justify this assumption with the calculation in an appendix.
15. L290 - introduction to Netherton syndrome could come earlier in the paper. This is quite a strong motivation for many of the modelling assumptions.
16. L314 this is a testable prediction. Is there any supporting evidence in the literature?
17. L380 'Similar results are seen to the diseased system' - I suggest avoiding use of 'healthy' and 'diseased'. It is a model, not a living tissue!
18. L510 - can the authors show the fit to the data.
19. L577 - Can the authors summarise the final model that couples to the mechanics. The main text would benefit from presentation of this model, especially the interpretation of Figure 2 in the results.
20. L 591 - I cannot find where η is defined in the text.
21. L591 - How are nearest neighbours defined?
22. L594 - The rotation force ought to be defined here for completeness.
23. Equations (19) - can the authors plot the force law for completeness and briefly explain its physical justification.
24. Rate parameter modifications - If increasing the proliferation rate is 'simply a rescaling of time', why do the authors not just modify the time step of the simulation? If the scaling is kept it would be clearer if it was performed more formally.
25. L636 - The output time step is now a model parameter and should be defined as such.
26. L647 - I suggest that the equation that describes how α depends on subcellular variables goes into the main text. This is one of the key assumption in the paper and is somewhat hidden in the Algorithm definition.

Minor typos

1. L29 flow - follow
2. L32 - missing molecules
3. the prose is overly colloquial in places - e.g. L 58 - the final pieces of the puzzle
4. L664 - density - number of cells per unit volume.
5. the authors use phrases such as ‘fill period’, ‘force-based cell removal’, ‘output time step’, ‘pull forces’ which to the non-initiated read like jargon. It would be better to explain what the principles are and then label them.
