# Supplementary material for: Multiscale modelling of desquamation in the interfollicular epidermis
Source: PLoS Comput Biol. 2022 Aug 29;18(8):e1010368. doi: 10.1371/journal.pcbi.1010368 (PMC9462764; doi:10.1371/journal.pcbi.1010368)
Supplement: Attachment — Submitted filename: ResponseToReviewers.pdf [file pcbi.1010368.s007.pdf]

Response to reviewers

We thank the reviewers for their useful critiques of the paper. We have responded to their comments and modified the paper accordingly and . Specifically, we have addressed each of their comments in the response below. Reviewers comments are numbered and shown in sans serif font, responses are given in Roman serif font and relevent extracts from the paper are given in *italics* where text that has been added in this revision is shown in green. Where line numbers are given they correspond to the revised version of the paper. (Note that due to the inclusion of tracked changes these line numbers don't correspond to the manuscript version of the revised manuscript with tracked changes).

Reviewer #1

In this paper Miller et al. present a computational model of epidermal tissue. The model is used to investigate how the thickness of the stratum corneum can be related to subcellular regulation of adhesion. The model is applied to a condition known as Netherton syndrome, a condition in which the removal of cells from the top-most layers is affected.

1. The introduction can better critique the state-of-the-art in the field so that the novelty and importance of the proposed model can be assessed.

We have expanded our review on current multiscale models of the epidermis, with a particular focus on approaches to modelling desquamation (note this includes the phenomonological model of desquamation by Ohno et al. which was published in December 2021, after the submission of this paper) and included some additional (non-epidermal) references (Page 4, Lines 91–147):

- *A common approach in multiscale OS models of epithelia is the inclusion of a subcellular ODE model for each cell [23-26]. For the epidermis, this approach has been used to investigate, for example, the role of TGF-β (transforming growth factor β: a signalling molecule thought to help regulate division and differentiation) in wound healing using a mass action model [23,24]. Another example is Kobayashi et al. [29] who used a phenomenological subcellular ODE model of calcium waves, from Kobayashi et al. [28], to understand calcium's role in epidermal homeostasis.*

  Another subcellular approach, also used to study the calcium gradient in the epidermis, is a molecular exchange model. Such a model incorporates the exchange of water and accompanying molecules, such as calcium, between adjoining cells. Grabe and Neuber [27] first implemented this in a two dimensional multiscale model and Sütterlin et al. [25] extended it to a three dimensional ellipsoidal model, to show such a system could maintain the calcium gradient itself and generate realistic tissue morphologies.

  Three dimensional ellipsoid/spheroid, rather than spherical, models have been used by Sütterlin et al. [25] and Ohno et al. [26]. In these models, a cell changes its shape (flattens) as it enters a new layer of the tissue. Though such ellipsoidal models would be expected to produce more realistic packings and morphologies, there is a trade off with computational time. Additionally, these models do not account for cell rotation in their implementations, which one might expect to cause changes in packing, particularly at the interface between the different layers when there is shape discontinuity.

  An alternative multiscale approach that has been used for the epidermis is a multicellular-extracellular model. For example, Schaller and Meyer-Hermann [32] included an extracellular reaction-diffusion model of nutrients and water to regulate division timing to investigate the persistence of melanoma cells in the epidermis under different parameter combinations [32].

A previously published multiscale model that incorporates subcellular dynamics for the adhesion degradation, and hence desquamation, is that of Ohno et al. [26]. The subcellular model is a phenomenological model to imitate the dynamics of the subcellular system described above. Desquamation of cells occurs when adhesion levels of the cell drops below a specified threshold (and has less than 16 cell neighbours). The authors include a deformable dermis layer in the model to understand the impact of the dermis on epidermis morphology. The study investigates homeostasis of the system, including tissue thickness, by modifying proliferation rates and stiffness of the dermis. This is in contrast to the results in this paper, in which we keep the proliferation rate constant and focus instead on changes to the desquamation process.

The difference between the desquamation model developed by Ohno et al. [26] and the model used in this paper is that Ohno et al. used a phenomenological model with parameters chosen by the authors. In contrast, our model is mechanistic, using enzyme kinetics models, and fully parameterised using data from the experimental literature, allowing us to more deeply interrogate the effects of the molecular reactants. Additionally, the use of an upward force for the cell removal is a way to investigate how cells at the surface could interact with their environment, i.e. removal in squames, and the balance between the degradation rate of adhesion and experienced environmental forces. The importance of environmental force in desquamation was demonstrated by Goldschmidt and Kligman [33] who observed skin cell accumulation when participant's skin was covered by a cup for 6 weeks.

Three multiscale models that incorporate simpler (non ODE) models for adhesion degradation are Li et al. [34], Schaller and Meyer-Hermann [32], and Sütterlin et al. [25]. Both Schaller and Meyer-Hermann [32] and Sütterlin et al. [25] degrade the adhesion between cells according to the cell age and time since differentiation respectively. In both models, cells are removed using an adhesion threshold, making removal dynamics similar to methodologies based on cell removal at a specified age. Li et al. [34] add an upward force to the Schaller and Meyer-Hermann model [32] to pull cells from the tissue in order to separate and remove them, which is similar to the mechanics we implement (described in the Model section). None of these three models investigate the molecular dynamics causing the adhesion degradation.

2. Can the authors better identify what the aims of the work and try to focus the emphasis of the results towards addressing particular aims.

We have clarified the aims of the work and focused the results in several places in the paper:

1. In the abstract:
   - *In this paper we will investigate combining a (mass action) subcellular model of desquamation with a three dimensional (cell centre based) multicellular model of the interfollicular epidermis to better understand maintenance of epidermal thickness. Specifically, our aim is to determine if a hypothesised biological model for the degradation of cell-cell adhesion, from the literature, is sufficient to maintain a steady state tissue thickness. These investigations show the model is able to provide a consistent rate of cell loss in the multicellular model.*
   - *A hypothetical treatment result is also investigated: we compare the cases of a partially effective homogeneous treatment (where all cells partially recover) and a totally effective heterogeneous treatment (in which a proportion of the cells totally recover) with the aim to determine the difference in the response of the tissue to these different scenarios. Results show an increased benefit to corneum thickness from the heterogeneous treatment over the homogeneous treatment.*

2. In the introduction:

   - Page 2, Lines 7–9: *The behaviour we focus on in this paper is desquamation, with the aim to improve our understanding of how it is controlled by subcellular processes in order to maintain tissue thickness.*
   - Page 5, Lines 153–163: *In this study, we implement the hypothesised subcellular biology of desquamation, described above and shown schematically in Figure 1, using a mass action model. The goal of the study is to determine whether the hypothesised model is sufficient to cause desquamation in the tissue and, in combination with a maintained proliferation rate, maintain a steady state tissue thickness. We show the results for this model at the subcellular scale for a single cell, highlighting the dynamics occurring as a cell migrates through the epidermis. We then investigate the desquamation process and epidermal homeostasis with a coupled subcellular-multicellular model, in both normal and abnormal (NS) scenarios, with the goal of determining how the tissue thickness is maintained, and how it responds to changes in inhibitor concentrations.*

3. For each of the results sections:

   - Page 6, Line 166: *The subcellular model is developed for use in a multiscale model of the epidermis, however first we consider the subcellular response of a single cell moving through a pH gradient. The goal of this study is to determine whether the derived ODEs and parameters, calculated from experimental literature, result in a degradation rate of the adhesion proteins commensurate with the expected migration time of the cell. A mass action enzyme kinetics model, with competitive inhibition, is developed for the interactions between KLK, LEKTI, and corneodesmosome (CND), as shown in Figure 1, to predict the proportion of CND remaining for the cell.*
   - Page 8, Lines 223–228: *For our multiscale model of the epidermis we couple the ODE system for subcellular desquamation with a multicellular model of the epidermis. This will allow us to investigate how events happening at a subcellular scale impact tissue dynamics—our aim is to determine whether subcellular reactions hypothesised in the literature can produce a homeostatic tissue thickness. Developing insights into how the scales interact is critical to developing a more comprehensive understanding of the tissue.*
   - Page 12, Lines 348–355: *Netherton Syndrome (NS) is a disorder that mutates the gene for the LEKTI inhibitor, reducing LEKTI concentrations in the epidermis [13]. Presumably as a result of the reduced amount of inhibitor, KLK levels are elevated in NS patients. Komatsu et al. [13] recorded KLK levels in NS patients to be between 157% to 486% that of healthy corneum. In order to simulate the effects of NS we can extend our model to consider the effect of reduced LEKTI on the tissue by reducing the concentration of the inhibitor,* i_T*. The goal of this study is to determine how the tissue thickness is related to the proportion of available inhibitor in the model.*
   - Page 13, Lines 414–424: *A multiscale multicellular model is well suited for investigating heterogeneous systems. If we consider the system above for abnormal tissue, we could hypothesise that a treatment to restore the system to its normal homeostasis would be to increase the level of inhibitor in the cells. The efficacy of this hypothetical treatment can be seen above in. Figure 5b where results show a linear relationship between the inhibitor concentration in the cells and the tissue thickness. However, one could also envisage a scenario in which such a treatment did not have 100% cell uptake, instead treating only a proportion of stem cells and leaving the remainder with reduced inhibitor levels. We investigate this scenario here with the goal of comparing change in tissue thickness when cells in the system have heterogeneous inhibitor levels to tissue thickness with homogeneous inhibitor levels.*

3. Details of the model equations need to provided in the main text.

We have ensured that all model equations are included in the main text. This is described below in response to the reviewer's specific comments (Comments 8 and 13).

4. Some of the simplifications made to make the model more efficient to simulate seem rather ad hoc. How do the authors know that the simplification are not affecting results in unanticipated ways? Is it worth simulating the model in 3D if one has to make such compromises? Are such simplifications made in the other models?

The majority of the simplifications made here are based off previously used modelling assumptions. With respect to the reduction in size, and time, of the system this is also a common way to reduce the computational requirements of the system (see epidermal models references [23],[24],[32]). With respect to proliferation time particularly, Adra et al. [24] found similar results for cell population numbers (per cell type) in their system when proliferation times were increased to 10 times the base level. The only novel simplification used here is the use of reduced concentration of enzyme instead of detailed modelling of the spatial diffusion of the reactants in the subcellular model. This does indeed have some effect on the model results, namely, as we state in the paper, we reduce the observed effect of the reduction in inhibitor (Page 13, Lines 381–385):

- *In Supplementary S1 Text (Section A2.4) we also find the use of an effective enzyme concentration decreases the effect of a reduction in inhibitor on total free enzyme when solving the subcellular model for a single migrating cell. Consequently, the 28% thickness reduction is expected to be a lower bound for the effect of the reduced inhibitor on tissue thickness.*

For the purposes we have used this model for (investigating how it interacts with the full tissue), we believe this simplification is reasonable. There may in fact be further effects of this, but we believe we have explicitly stated the limitations of this model and, currently, limitations in detailed data would make a more detailed model difficult to derive and implement.

With respect to using a 3D system, the main advantage is the lack of regular packing of the cells which can introduce artefacts (see ref. 39 in paper: Osborne et al., doi:10.1371/journal.pcbi.1005387). In particular for this system, the use of a 2D system with regular packing would cause the cells to peel off in layers (preliminary results in 2D showed this). We have added a sentence to the paper, in the Model section, on this (Page 24, Lines 679–684):

- *We use a modified version of the three dimensional multicellular model in Miller et al. [35]. This model uses an overlapping spheres methodology, where cells interact with each other via adhesion and repulsion forces, and with the basal membrane. A three dimensional model is used to avoid unnecessary artefacts of two dimensional hexagonal packing which can cause synchronisation of cell removal due to geometric constraints.*

5. L2 Are all experimental data taken from human studies?

The reviewer is referring to the sentence: *The thickness of the epidermis varies both between body location and individual, ranging from 50 to 100 μm [1,2].* This is experimental data from human studies, and we have clarified this in the text by changing the sentence (Page 2, Lines 2–3) to:

- *The thickness of the epidermis varies both between body location and individual, with averages in the range 50 to 100 μm measured in human studies [1,2].*

6. L103 Can the authors expand on why it is necessary to model subcellular processes controlling adhesion degradation? What scientific question is addressed by doing this? Perhaps this point can be addressed in the discussion.

The reason for modelling subcellular processes is to better understand the connection between what is occurring at a subceullar level and the response at a tissue level. Given that dysfunction in systems will often be due to processes occurring at a subcellular scale, developing models that can provide insight into how the scales interact is very useful in understanding better both the normal system and the system with dysfunction. Specifically for the epidermis, the desquamation process is critical to the health of the tissue and hence a full systems model, incorporating all scales, we believe will be critical to answering many of the currently open questions about the tissue.

We have added details on the question we are answering in the results section the reviewer has referenced (Page 8, Lines 223–228):

- *For our multiscale model of the epidermis we couple the ODE system for subcellular desquamation with a multicellular model of the epidermis. This will allow us to investigate how events happening at a subcellular scale impact tissue dynamics—our aim is to determine whether subcellular reactions hypothesised in the literature can produce a homeostatic tissue thickness. Developing insights into how the scales interact is critical to developing a more comprehensive understanding of the tissue.*

We have also added further clarification in the discussion (Page 18, Lines 486–495):

- *In this paper we have introduced a multiscale model for desquamation of the epidermis. The coupling of the two scales allows us to investigate whether the subcellular model is sufficient to maintain desquamation at a tissue level, and how the tissue responds to changes to subcellular parameters. In Figure 4 we showed that the coupled model is able to maintain a homeostatic tissue height. The height of the tissue is determined by the balance between the proliferation rate and the desquamation rate. The system with reduced LEKTI in the subcellular model (abnormal tissue) has an increased desquamation rate, changing this balance and hence the thickness of the tissue. Consequently, we can use such a multiscale system to investigate the effect of different aspects of proliferation and desquamation on tissue thickness.*

The clarification of study aims in response to Comment 2 has also added further context for the use of a coupled model, such as the following additions to the introduction:

- Page 2, Lines 7–9: *The behaviour we focus on in this paper is desquamation, with the aim to improve our understanding of how it is controlled by subcellular processes in order to maintain tissue thickness.*
- Page 5, Lines 153–163: *In this study, we implement the hypothesised subcellular biology of desquamation, described above and shown schematically in Figure 1, using a mass action model. The goal of the study is to determine whether the hypothesised model is sufficient to cause desquamation in the tissue and, in combination with a maintained proliferation rate, maintain a steady state tissue thickness. We show the results for this model at the subcellular scale for a single cell, highlighting the dynamics occurring as a cell migrates through the epidermis. We then investigate the desquamation process and epidermal homeostasis with a coupled subcellular-multicellular model, in both normal and abnormal (NS) scenarios, with the goal of determining how the tissue thickness is maintained, and how it responds to changes in inhibitor concentrations.*

7. L 111-127 - I did not find this overview helpful and suggest removing.

The reviewer is referring to the introductory paragraphs in the results section. We appreciate the reviewer's feedback on this and have removed the paragraph as suggested.

8. L131 - present the model equations. One of key proposed novelties of the paper is a model for desquamation. In L148 the rate parameters k_1 and k_2 are discussed but these have not been introduced in the main text.

We have added the model equations to this section as suggested by the reviewer this includes definition of model parameters (Page 6, Lines 166–176):

- *The subcellular model is developed for use in a multiscale model of the epidermis, however first we consider the subcellular response of a single cell moving through a pH gradient. The goal of this study is to determine whether the derived ODEs and parameters, calculated from experimental literature, result in a degradation rate of the adhesion proteins commensurate with the expected migration time of the cell. A mass action enzyme kinetics model, with competitive inhibition, is developed for the interactions between KLK, LEKTI, and corneodesmosome (CND), as shown in Figure 1, to predict the proportion of CND remaining for the cell. We define $[S], [I], [E], [C_S], [C_I]$, and $[P]$ as the concentrations of free substrate CND, free inhibitor LEKTI, free enzyme KLK, KLK-CND complex, KLK-LEKTI complex, and CND degradation products respectively. We can then define dimensionless variables $s = [S]/s_0$; $i = [I]/s_0$; $e = [E]/e_T$; $c_s = [C_S]/e_T$; $c_i = [C_I]/e_T$; and $p = [P]/s_0$ for some initial concentration of the substrate, s_0, and total amount of enzyme in the system, e_T. This gives the system of ODEs for the degradation of the adhesion as follows:*

$$\frac{\mathrm{d}e}{\mathrm{d}t} = -k_{+1}s_0es + (k_{-1} + k_2)\, c_s - k_{+3}s_0ei + k_{-3}c_i\,, \tag{1}$$

$$\frac{\mathrm{d}s}{\mathrm{d}t} = -k_{+1}e_Tes + k_{-1}\frac{e_T}{s_0}c_s\,, \tag{2}$$

$$\frac{\mathrm{d}i}{\mathrm{d}t} = -k_{+3}e_Tei + k_{-3}\frac{e_T}{s_0}c_i\,, \tag{3}$$

$$\frac{\mathrm{d}c_s}{\mathrm{d}t} = k_{+1}s_0es - (k_{-1} + k_2)\, c_s\,, \tag{4}$$

$$\frac{\mathrm{d}c_i}{\mathrm{d}t} = k_{+3}s_0ei - k_{-3}c_i\,, \tag{5}$$

$$\frac{\mathrm{d}p}{\mathrm{d}t} = k_2\frac{e_T}{s_0}c_s\,. \tag{6}$$

  A full derivation of the model is given in the Model section, and parameters are given in Table 1.

  The model uses the cell's vertical location to determine its local pH. We define $\xi \in [0, 1]$ as the vertical dimension normalised by steady state corneum height with origin ($\xi = 0$) at the interface between the corneum and the lower epidermis (here we define the interface to be at $z = 4$). For any cells above the determined corneum height we set $\xi = 1$. The cell's local pH is then determined as a function of ξ:

$$\mathrm{pH} = f_{\mathrm{pH}}(\xi) = 6.8482 - 0.3765\,\xi - 5.1663\,\xi^2 + 3.1792\,\xi^3\,. \tag{7}$$

  Using this, the rate parameters for the KLK-LEKTI interaction, k_{+3} and k_{-3}, are determined:

$$k_{+3} = f_{+3}(\mathrm{pH}) = (5.2\,pH - 19.5) \times 10^7\ [M^{-1}.hr^{-1}], \tag{8}$$

$$k_{-3} = f_{-3}(\mathrm{pH}) = 2.3 \times 10^6\, e^{-3.0pH}\ [hr^{-1}]. \tag{9}$$

The proportion of remaining adhesion proteins (CND) is the output used by the multicellular system for the multiscale model. Further details on these functions are given in the Model section, and in Supplementary S1 Text (Section A1).

9. L141 - is the constant velocity assumption valid (check by comparing with simulation results). I would expect to see a velocity gradient along the z axis.

The reviewer is referring to the line (in the original paper): *We assume that the cell velocity is approximately constant through the stratum corneum, and estimate the migration time as 20 days [33].* We wish to emphasise that we are only interested in the velocity when the cell is in the stratum corneum layer—we would not assume this to hold throughout the whole of the tissue.

With respect to model results, velocity varies within the first few layers of the tissue, where cells are close to the proliferation zone and hence they are responding to local division events and are acelerating upwards. Once cells enter the higher layers of the tissue, the stratum corneum, we find that they appear to stabilise to a reasonably constant vertical velocity. We have calculated the variance of velocity of cells within the stratum corneum and found it to be, on average, 5.7% (as we have added to the text). Consequently we believe this is a valid assumption.

Physically, we would also expect this to be the case within the stratum corneum, given the cells in this layer have reached their final shape and adhere to each other in layers. Given a constant proliferation rate in the basal layer, one could hypothesise that the velocity would be roughly constant in these upper layers.

We have stated the basis of this assumption in the text by modifying the text as follows (Page 7, Lines 177–183):

- *We investigate the model dynamics for a single cell moving upwards through the corneum by assuming that the cell velocity is approximately constant through the corneum. This assumption is supported by results from our simulations where the variation in a cell's (daily) velocity as it migrates through the corneum is, on average, 5.7% (for the normal tissue described below). If we assume a migration time of 20 days [36] the approximate upwards velocity, normalised by corneum thickness, is* $v_\xi = 0.05\ \text{day}^{-1}$.

10. L147 - expand on the results here.

The reviewer is referring to the following line from the original text: *Results showed the system parameters determined using the literature produced desquamation significantly faster than expected (see S1 Text for these results).*

We have expanded on the results as requested (Page 7, Lines 184–193):

- *Results (shown in Supplementary Figure S5) showed the calculated system parameters, determined using* in vitro *and* in vivo *data from the literature, degraded the adhesion proteins significantly faster than expected: the corneodesmosome, s, degrades within a day ($s = 0.009$ at $t = 24$ hr), rather than the expected time of 20 days (the migration time of the cell [36]). Degradation rates of s over this 24 hour time period reach a maximum of $0.13\ \text{hr}^{-1}$ (at $t = 2$ hr), despite the high level of enzyme that is in complex with the inhibitor: $c_i = 0.996$ on average for $t \leq 24$ hours. These high rate values are due to the high effective rate parameter for complex*

formation in Equation (17): $k_{+1}e_T = -49.7\ \text{hr}^{-1}$. This indicates that either the rate parameters are wrong (k_{+1} and k_2), or that the mechanism is wrong.

11. L137 - the authors could outline the main equations in the results section.

We have outlined the main equations in the results section, as detailed in the response to Comment 8.

12. L172 - by what factor is eT reduced. This is not mentioned in the figure caption and not obvious in the appendix. Given the uncertainties in parameter estimates is it necessary to go into such detail about the parameter fitting here. This section detracts from the flow of the paper. Perhaps use a lower value and note this is the case. Then in the discussion go into some more detail, highlighting potential mechanisms that could results in an effective parameter.

We thank the reviewer for pointing out this detail is missing. We have added this information both in the caption and the text:

- Page 8, Caption Fig 2: *Solution of the subcellular model for a cell moving at a specified velocity through the pH gradient of the corneum, using an effective concentration of KLK enzyme: $s_0 = 10\ \mu M$, $e_T = i_T = 0.1\ nM$. This is a reduction in enzyme by a factor of 10^{-4} (from $0.7\ \mu M$).*
- Page 7, Lines 211–214: *Figure 2 shows the system dynamics with an effective concentration of e_T, reduced from $0.7\ \mu m$ to $0.1\ nm$. The figure shows that the effective concentration is able to reproduce the rates expected for the desquamation process: the majority of s degrades by T=20 days, which is the expected migration time of a cell [36].*

We have also moved the detail from the results to the discussion as requested. The following summary has been left in the results section (Page 7, Lines 201–210):

- *By limiting the diffusion of the enzyme, the system is no longer homogeneously mixed and consequently the mass action model does not hold. A simple, and computationally efficient, way to counteract the effect of limited diffusion of the enzyme is to reduce its concentration, which reduces the effective rate parameter ($k_{+1}e_T$) in Equation (17) for degradation of s. The use of an effective concentration of enzyme is somewhat similar to using an effective volume fraction for crowded macromolecular diffusion [34]. For simplicity, we also keep $i_T = e_T$, as limited diffusion would have a similar affect on the inhibitor. A full investigation into the desquamation rate and the effect of the reactant concentrations is given in S1 Text (Section A2).*

and we have moved the detailed description to the discussion section (Page 18, Lines 472):

- *Macromolecular crowding is a known phenomenon in biological media generally, and modelling the resulting diffusion dynamics is a very complex problem [37,39]. In order to compensate for this limited diffusion effect, without incorporating complex new processes in the model, we instead propose the use of an effective concentration of enzyme. Limited diffusion would reduce the amount of available enzyme to bind to the substrate. Decreasing e_T has the same effect as it directly affects the effective rate parameter for the degradation of s ($k_{+1}e_T$). Consequently this is a way to simulate limited diffusion in a computationally efficient way. We maintain $i_T = e_T$, as limited diffusion would also affect the inhibitor. Figure 2 shows a reduced concentration of e_T is able to reproduce the rates expected for the desquamation process (on the scale of 20 days [36]).*

13. L174 - provide a brief introduction to the multicellular model here (e.g. equations of motion, boundary conditions, cell cycle rules provide a table with parameter values in the main text etc). These details are crucial for the interpretation of the main results. Special focus ought to be paid to how the molecular model couples to the adhesion term in the multicellular model.

The model is fully described in "The multiscale model" section in Methods, and all multicellular parameters are given in Table 3. We have added a brief description on the multicellular model to the results section, with a focus on the coupling between the two scales. However we have chosen keep full technical. details in the methods section to maintain the flow of the paper. The added text is as follows (Page 8, Lines 229–246):

- *The multicellular model is an overlapping spheres model, with cell movement calculated using the inertia-less force balance:*

$$\eta \frac{d\mathbf{c}_i}{dt} = \sum_{j \in N_i} \mathbf{F}_{ij} + \mathbf{F}_i^{\mathbf{Rt}} + \mathbf{F}_i^{\mathbf{D}} \,, \tag{10}$$

where $\mathbf{c}_i$ is the cell centre location of cell i; N_i is the set of neighbours of cell i; $\mathbf{F}_{ij}$ are the forces on cell i due to interacting neighbour cell j; $\mathbf{F}_{i,ext}$ are any external forces acting on cell i; and η is the viscous drag coefficient for the cell [38]. We define $\hat{\mathbf{r}}_{ij}$ as the unit vector between cells i and j and $r_{ij} = \|\mathbf{c}_j - \mathbf{c}_i\| - R_{ij}$ where R_{ij} is the sum of the cell radii (1 CD except when cells are dividing). The force of interest for the coupling between the models is the adhesion force between two cells, $\mathbf{F}_{ij}^{\mathbf{A}}$ (this is $\mathbf{F}_{ij}$ for $r_{ij} > 0$, see Equation (31)). This force is based on the function used by Li et al. [34]:

$$\mathbf{F}_{ij}^{\mathbf{A}} = -\alpha_{ij} \left(\left(r_{ij}^* + c \right) e^{-\gamma \left(r_{ij}^* + c \right)^2} - c e^{-\gamma \left(r_{ij}^{*2} + c^2 \right)} \right) \hat{\mathbf{r}}_{ij}, \; r_{ij} > 0, \qquad \text{where } c = \sqrt{\frac{1}{2\gamma}}, \tag{11}$$

$$\text{and } r_{ij}^* = \frac{r_{ij}}{R_0}, \tag{12}$$

where α_{ij} is the adhesion coefficient between the two cells which is scaled by their CND level (s) from the subcellular model as described below, and $R_0 = 0.5$ cell diameters is the normal radius of a cell. A full description of all other forces in the model is given in the Model section.

The cell's vertical location in the corneum in the multicellular system, i.e. ξ (acting as a proxy for pH), provides the input into the subcellular model. For each cell, at each multicellular timestep, the subcellular model is integrated over the duration of the multicellular model time step to determine the cell's updated CND proportion (s_i). The new proportion of CND (s_i) is returned to the multicellular system and is used to scale the magnitude of the cell's adhesion to its neighbours using the average of the two cell's CND levels:

$$\alpha_{ij} = \frac{1}{2}(s_i + s_j)\alpha_0 \,, \tag{13}$$

where α_{ij} is the adhesion coefficient in Equation (11) between cells i and j and α_0 is the normal cell-cell adhesion coefficient. A diagram to illustrate this coupling is shown in Figure 3. As we explain in the Model section, given the increased proliferation rate used in the model, the rate parameters for the multicellular system are scaled in order to reduce the height of the system for computational efficiency (Table 1).

14. L191 Critique comparison with ref. [131] in the discussion section.

We assume here the reviewers are referring to reference [31] (now [35]). The model in [31] is a precursor to this paper and details the mechanics of the multicellular model we use in this paper, except the

mechanics of desquamation—it uses a sloughing model (cells removed at a certain height). This paper extends the model from [31] to add the subcellular model for desquamation, as well as further mechanics to support this. We have added further detail in the text (Page 9, Lines 252–256):

- *A more detailed description of the multicellular model, including the coupling and desquamation model, is provided below in the Model section. This model extends the (purely mechanical) model from Miller et al. [35] by incorporating the novel chemical subcellular model for adhesion degradation, and additional mechanics to model the resulting desquamation dynamics (as opposed to the sloughing model used in [35]).*

15. L194 - remove healthy!

We have removed this term as suggested, the sentence now just says *to produce a homeostatic tissue.*

16. L201 - what system?

The word system has been replaced with tissue (and, in the next sentence, model) for clarity (Page 9, Lines 263–267):

- *The outcome of interest for the multiscale model is the homeostatic corneum, and hence tissue, thickness. Figure 4b shows the mean thickness of the tissue over time and the estimated steady state thickness of the tissue and the corneum. We can see that this new model is able to maintain a steady state thickness. The steady state tissue and corneum thickness in Figure 4b is 14.8 CD and 10.8 CD respectively.*

17. L211-L225 the description of the results in the text is overly numerical. What is the significance of the described numbers? This section could be reduced, with the focus being on the key principles (emergence of homeostasis and dependency on key parameters).

We have modified the text as follows (Page 10, Lines 275–296):

- *In order to better understand the maintenance of tissue thickness, and the interaction between the two scales, we analyse the dynamics of the subcellular model. The levels of the reactants over tissue thickness for one realisation, at $t = 30$ days, are shown in Figure 4c. The point data shows the multicellular simulation values, while the lines indicate the expected values using the single cell solution. These expected values are determined, as in Figure 2, using the mean velocity of cells in the multicellular system (shown in Figure 4d). This calculation only includes the 'migratory cells': cells that are not proliferative cells or surface cells (those experiencing the removal force).*

  Figure 4c shows little variation in the reactant concentrations, up until the steady state thickness (dotted line), in cells at any particular height (s varies by $\pm 1.5\%$ on average). This can also qualitatively be observed in Figure 4a. Consequently, for a maintained proliferation rate, the tissue reaches a homeostatic state in which reactant levels are invariant in the horizontal directions and, given this, a cell's reactant levels at any location can be well-approximated by solving for a single migrating cell using the emergent cell velocity in the multicellular system. This causes a maintained homeostatic desquamation rate at the tissue level, and consequently a maintained tissue thickness.

Figure 4c also shows significant divergence in the levels above the steady state thickness (most significantly for s, the green points in Figure 4c). This divergence is an effect of the removal force applied to a surface cell when it is in the top layer of the tissue. This force causes higher velocities for surface cells compared to the velocities of migratory cells in the bulk of the tissue.

18. L259 - this section is drifting somewhat from the main focus of the paper and seems some- what incidental. Could the authors set the model up with a single proliferating cell population and justify this assumption with the calculation in an appendix.

The reviewer is referring to the section about approximating two proliferative populations with a single proliferative population. We have moved this section into the supplementary text and it is now just summarised as a secondary result in the previous section (Incorporation of the subcellular model of adhesion into a multicellular model allows the study of desquamation). The text moved to the previous section is as follows (Page 12, Lines 329–345):

- *Experiments have shown evidence of two populations of proliferative cells in the basal layer with different cell cycle lengths [5-7]. We can investigate the effect of two proliferative populations in our model by comparing different combinations of fast and slow cycling proliferative cell populations with the same harmonic mean:* $H(\mathrm{T_C}) = 15$ *hours. The harmonic mean ensures the overall proliferation rate is the same across each setup, as it accounts for the fact that fast cycling cells undergo more cycles than slow cycling cells. Due to this behaviour, we would expect an increased number of divisions to occur than would be inferred from the arithmetic mean. We also ran simulations using the same arithmetic mean which confirmed this.*

  To implement the two populations, we set 50% of the cells as fast cycling: $\mathrm{T_C} = \mathrm{T_1}$*, and 50% as slow cycling:* $\mathrm{T_C} = \mathrm{T_1} + \Delta\mathrm{T_C}$*. Simulation results and a detailed analysis are given in Supplementary S1 Text (Section A3). Results show increasing the cycle difference,* $\Delta\mathrm{T_C}$*, but maintaining the same harmonic mean produces the same average proliferation rate and hence average* z *velocities. Consequently, the steady state corneum thickness is also unchanged. From this study, we know we can approximate the expected steady state thickness of a system with two populations of proliferative cells by a single population system with cycle time equal to the harmonic mean of the two populations.*

19. L290 - introduction to Netherton syndrome could come earlier in the paper. This is quite a strong motivation for many of the modelling assumptions.

We have moved some of the introduction to Netherton Syndrome to the introduction section:

- Page 3, Lines 30–35: *Diseases of the skin can range from a nuisance to a major health risk, with debilitating or even fatal consequences, and even the milder diseases can have follow on effects for our health. One such disease, which we use as an example in this paper, is Netherton Syndrome (NS). This disease affects the subcellular desquamation process, causing premature desquamation, skin peeling, frequent skin infection, and temperature instability [20].*
- Page 3, Lines 77–81: *This subcellular system is disrupted in NS patients. The gene for the LEKTI inhibitor is mutated and, as a result, the concentration of LEKTI in the system is reduced. This is thought to be the reason NS patients have higher levels of KLK in the system, and is expected to cause an increase in the degradation rate of the cell's adhesion proteins [20].*

20. L314 this is a testable prediction. Is there any supporting evidence in the literature?

We are not aware of any studies measuring the thickness of patients with Netherton Syndrome, however there is a study that has been done measuring the amount of KLK enzyme in their system. We already use this in our Supplementary Results, but have added further text to the main paper on this (Page 12, Lines 370–385):

- *Figure 5b also shows that corneum thickness, τ_{ss}, is linearly dependent on the concentration of inhibitor present in the system. A linear fit is given on the plot. When there is no inhibitor present the corneum thickness decreases by 28%. Though we have no experimental data for reduction in corneum thickness for NS patients, we can make a comparison with data from Komatsu et al. [13] using the amount of free KLK in the system. The study found that NS patients had KLK levels 157%–486% that of healthy stratum corneum. We can calculate the amount of free enzyme in the system by taking the sum of the level of e for all cells in the corneum, up to the steady state thickness (we use the average over time). Comparing this for the normal and abnormal model results (Figure 4c and Figure 5c), the abnormal tissue with no inhibitor has 190% the free enzyme of the normal system—within the bounds of the observed increase, but towards the lower end despite being the worst case scenario. In Supplementary S1 Text (Section A2.4) we also find the use of an effective enzyme concentration decreases the effect of a reduction in inhibitor on total free enzyme when solving the subcellular model for a single migrating cell. Consequently, the 28% thickness reduction is expected to be a lower bound for the effect of the reduced inhibitor on tissue thickness.*

21. L380 'Similar results are seen to the diseased system' - I suggest avoiding use of 'healthy' and 'diseased'. It is a model, not a living tissue!

We have changed the terminology to 'normal' and 'abnormal' tissue/cells. We have also defined this in the Results in the text (Page 12, Lines 356–358):

- *We define normal tissue as tissue where cells release normal levels of inhibitor, $i_T = e_T$ for all cells, and abnormal tissue as tissue where cells have reduced levels of inhibitor, $i_T < e_T$.*

22. L510 - can the authors show the fit to the data.

Given the current length of the paper, we think, for brevity, it is not useful to explicitly include the plots in the main text, particularly as we believe this would necessitate adding further plots for all the parameter fits used in the model. We have however made the reference to the supplementary figure detailing the fits more explicit in the text so it is easier for the reader to find in the supplementary materials (Page 21, Lines 600–602):

- *A linear regression (using a log transform for k_{-3}) on this data produces the following equations for k_{+3} and k_{-3} as functions of pH:*

$$k_{+3} = f_{+3}(\text{pH}) = (5.2\,pH - 19.5) \times 10^7 \; [M^{-1}.hr^{-1}], \tag{23}$$

$$k_{-3} = f_{-3}(\text{pH}) = 2.3 \times 10^6 \, e^{-3.0pH} \; [hr^{-1}], \tag{24}$$

with R-squared values of $R^2 = 0.94$ and $R^2 = 0.9998$ respectively. These fits are plotted in Supplementary Figure S3.

23. L577 - Can the authors summarise the final model that couples to the mechanics. The main text would benefit from presentation of this model, especially the interpretation of Figure 2 in the results.

We have added further detail on the models and coupling to the results section, as detailed in response to Comment 8 and 13. Given the final model is now summarised in the Results, as well as being fully detailed in the Model section, we have not added another summary in the Model section. The following paragraph has also been added to help with interpretation of Figure 2 (Page 7, Lines 211–220):

- *Figure 2 shows the system dynamics with an effective concentration of e_T, reduced from 0.7 μm to 0.1 nm. The figure shows that the effective concentration is able to reproduce the rates expected for the desquamation process: the majority of s degrades by T=20 days, which is the expected migration time of a cell [36]. The dynamics of the system are strongly dependent the level of c_i which determined by the ratio of k_{+3} to k_{-3}, which varies with pH. More of the enzyme in complex with the inhibitor means there is less enzyme actively degrading the adhesion proteins (s), slowing the desquamation rate. These results support the hypothesis that the pH gradient in the skin is critical to controlling the rate of desquamation. This can also be seen in Supplementary Figure S4 where we solve the system with constant pH levels.*

The model summaries, as detailed in Comments 8 and 13, are:

- Page 6, Lines 166–176: *The subcellular model is developed for use in a multiscale model of the epidermis, however first we consider the subcellular response of a single cell moving through a pH gradient. The goal of this study is to determine whether the derived ODEs and parameters, calculated from experimental literature, result in a degradation rate of the adhesion proteins commensurate with the expected migration time of the cell. A mass action enzyme kinetics model, with competitive inhibition, is developed for the interactions between KLK, LEKTI, and corneodesmosome (CND), as shown in Figure 1, to predict the proportion of CND remaining for the cell. We define $[S], [I], [E], [C_S], [C_I]$, and $[P]$ as the concentrations of free substrate CND, free inhibitor LEKTI, free enzyme KLK, KLK-CND complex, KLK-LEKTI complex, and CND degradation products respectively. We can then define dimensionless variables $s = [S]/s_0$; $i = [I]/s_0$; $e = [E]/e_T$; $c_s = [C_S]/e_T$; $c_i = [C_I]/e_T$; and $p = [P]/s_0$ for some initial concentration of the substrate, s_0, and total amount of enzyme in the system, e_T. This gives the system of ODEs for the degradation of the adhesion as follows:*

$$\frac{\mathrm{d}e}{\mathrm{d}t} = -k_{+1}s_0es + (k_{-1} + k_2)\,c_s - k_{+3}s_0ei + k_{-3}c_i\,, \tag{1}$$

$$\frac{\mathrm{d}s}{\mathrm{d}t} = -k_{+1}e_Tes + k_{-1}\frac{e_T}{s_0}c_s\,, \tag{2}$$

$$\frac{\mathrm{d}i}{\mathrm{d}t} = -k_{+3}e_Tei + k_{-3}\frac{e_T}{s_0}c_i\,, \tag{3}$$

$$\frac{\mathrm{d}c_s}{\mathrm{d}t} = k_{+1}s_0es - (k_{-1} + k_2)\,c_s\,, \tag{4}$$

$$\frac{\mathrm{d}c_i}{\mathrm{d}t} = k_{+3}s_0ei - k_{-3}c_i\,, \tag{5}$$

$$\frac{\mathrm{d}p}{\mathrm{d}t} = k_2\frac{e_T}{s_0}c_s\,. \tag{6}$$

A full derivation of the model is given in the Model section, and parameters are given in Table 1.

The model uses the cell's vertical location to determine its local pH. We define $\xi \in [0, 1]$ as the vertical dimension normalised by steady state corneum height with origin ($\xi = 0$) at the

interface between the corneum and the lower epidermis (here we define the interface to be at $z = 4$*). For any cells above the determined corneum height we set* $\xi = 1$*. The cell's local pH is then determined as a function of* ξ*:*

$$\mathrm{pH} = f_{\mathrm{pH}}(\xi) = 6.8482 - 0.3765\,\xi - 5.1663\,\xi^2 + 3.1792\,\xi^3\,. \tag{7}$$

Using this, the rate parameters for the KLK-LEKTI interaction, k_{+3} *and* k_{-3}*, are determined:*

$$k_{+3} = f_{+3}(\mathrm{pH}) = (5.2\,pH - 19.5) \times 10^7\ [M^{-1}.hr^{-1}], \tag{8}$$

$$k_{-3} = f_{-3}(\mathrm{pH}) = 2.3 \times 10^6\,e^{-3.0pH}\ [hr^{-1}]. \tag{9}$$

The proportion of remaining adhesion proteins (CND) is the output used by the multicellular system for the multiscale model. Further details on these functions are given in the Model section, and in Supplementary S1 Text (Section A1).

- Page 8, Lines 229–246: *The multicellular model is an overlapping spheres model, with cell movement calculated using the inertia-less force balance:*

$$\eta \frac{d\mathbf{c}_i}{dt} = \sum_{j \in N_i} \mathbf{F}_{ij} + \mathbf{F}_i^{\mathbf{Rt}} + \mathbf{F}_i^{\mathbf{D}}\ , \tag{10}$$

where $\mathbf{c}_i$ *is the cell centre location of cell* i*;* N_i *is the set of neighbours of cell* i*;* $\mathbf{F}_{ij}$ *are the forces on cell* i *due to interacting neighbour cell* j*;* $\mathbf{F}_{i,\mathrm{ext}}$ *are any external forces acting on cell* i*; and* η *is the viscous drag coefficient for the cell [38]. We define* $\hat{\mathbf{r}}_{ij}$ *as the unit vector between cells* i *and* j *and* $r_{ij} = \|\mathbf{c}_j - \mathbf{c}_i\| - R_{ij}$ *where* R_{ij} *is the sum of the cell radii (1 CD except when cells are dividing). The force of interest for the coupling between the models is the adhesion force between two cells,* $\mathbf{F}_{ij}^{\mathbf{A}}$ *(this is* $\mathbf{F}_{ij}$ *for* $r_{ij} > 0$*, see Equation (31)). This force is based on the function used by Li et al. [34]:*

$$\mathbf{F}_{ij}^{\mathbf{A}} = -\alpha_{ij}\left(\left(r_{ij}^* + c\right)e^{-\gamma\left(r_{ij}^*+c\right)^2} - ce^{-\gamma\left(r_{ij}^{*2}+c^2\right)}\right)\hat{\mathbf{r}}_{ij},\ r_{ij} > 0, \qquad \text{where } c = \sqrt{\frac{1}{2\gamma}}, \tag{11}$$

$$\text{and } r_{ij}^* = \frac{r_{ij}}{R_0}, \tag{12}$$

where α_{ij} *is the adhesion coefficient between the two cells which is scaled by their CND level (s) from the subcellular model as described below, and* $R_0 = 0.5$ *cell diameters is the normal radius of a cell. A full description of all other forces in the model is given in the Model section.*

The cell's vertical location in the corneum in the multicellular system, i.e. ξ *(acting as a proxy for pH), provides the input into the subcellular model. For each cell, at each multicellular timestep, the subcellular model is integrated over the duration of the multicellular model time step to determine the cell's updated CND proportion* (s_i)*. The new proportion of CND* (s_i) *is returned to the multicellular system and is used to scale the magnitude of the cell's adhesion to its neighbours using the average of the two cell's CND levels:*

$$\alpha_{ij} = \frac{1}{2}(s_i + s_j)\alpha_0\,, \tag{13}$$

where α_{ij} *is the adhesion coefficient in Equation (11) between cells* i *and* j *and* α_0 *is the normal cell-cell adhesion coefficient. A diagram to illustrate this coupling is shown in Figure 3. As we explain in the Model section, given the increased proliferation rate used in the model, the rate parameters for the multicellular system are scaled in order to reduce the height of the system for computational efficiency (Table 1).*

24. L 591 - I cannot find where η is defined in the text.

We thank the reviewer for noting this omission, η has been added to the parameter table (Table 1) and is described in the text when it is used (Page 8, Line 230 and Page 24, Line 688): *and η is the viscous drag coefficient for the cell...*

25. L591 - How are nearest neighbours defined?

Neighbours are defined to be any cell within a certain radius of the cell of interest. We define this radius to be 2 cell diameters. This has been added to the text (Page 24, Line 686–679):

- *where $\mathbf{c}_i$ is the cell centre location of cell i; N_i is the set of neighbours of cell i; $\mathbf{F}_{ij}$ are the forces on cell i due to interacting neighbour cell j; $\mathbf{F}_{i,\text{ext}}$ are any external forces acting on cell i; and η is the viscous drag coefficient for the cell [38]. A cell's neighbours are any cells within 2 cell diameters (distance between cell centres) of the cell of interest.*

26. L594 - The rotation force ought to be defined here for completeness.

We have added the definition of the rotation force (Page 24, Lines 703–708):

- *The rotational force, $\mathbf{F}_i^{\mathbf{Rt}}$ in Equation (30), between two daughter cells, i and j, during division is from Miller et al. [35] and is defined as:*

$$\mathbf{F}_i^{\mathbf{Rt}} = -k_\phi \phi \hat{\mathbf{n}}\,, \tag{34}$$

where k_ϕ is a torsional spring constant (see Table 1); ϕ is the angle (in radians) between the vertical direction, $\mathbf{k}$, and $\mathbf{r}_{ij}$; and $\hat{\mathbf{n}}$ is a unit normal to $\mathbf{r}_{ij}$ defined as:

$$\hat{\mathbf{n}} = \frac{\mathbf{r}_{ij} \times (\mathbf{k} \times \mathbf{r}_{ij})}{\|\mathbf{r}_{ij} \times (\mathbf{k} \times \mathbf{r}_{ij})\|}. \tag{35}$$

This force is used to maintain the stem cell population in the basal layer. For further detail see Miller et al. [35].

27. Equations (19) - can the authors plot the force law for completeness and briefly explain its physical justification.

We have added the following figure with the force as requested (Figure 8, Page 25 in the paper):

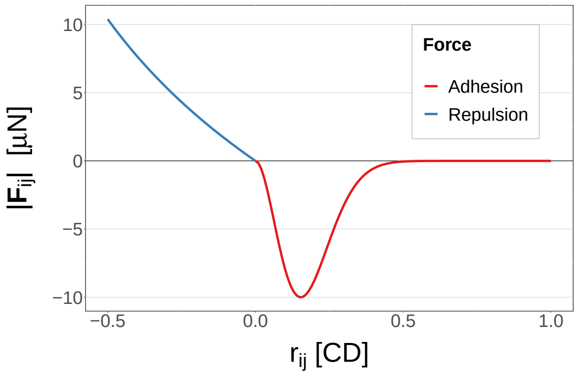

as well as the following explanation in the text (Page 24, Lines 697–702):

- *A plot of the adhesion and repulsion forces is shown in Figure 8. The adhesion force represents the effects of adhesion proteins between the cells: as the cells start to separate they experience forces from these proteins to pull them together, but as they separate further these adhesion proteins would begin to break apart. The repulsion force accounts for conservation of volume in the cells, and the steepness of its funciontion is chosen to avoid instability in the model [42,46].*

28. Rate parameter modifications - If increasing the proliferation rate is 'simply a rescaling of time', why do the authors not just modify the time step of the simulation? If the scaling is kept it would be clearer if it was performed more formally.

We appreciate the reviewer's enquiry about this. In mechanics based multicellular simulations numerical parameters are chosen to ensure the mechanics is stable. This is due to both proliferation and relaxation within the system. We are unable to increase the numerical time step of the simulation as we have kept the same mechanical parameters as in previous papers (references [31,37,38,41] for example), while there would be less proliferation the relaxation timescale is the same and hence we need to use the same numerical time step. Therefore we can increase the proliferation rate without having to decrease the timestep making simulations more computationally efficient.

The time step is now explicitly mentioned in the parameter table (Table 1, Page 22) and we have explained the necessity of keeping the same time step, from previous work, in the paper (Page 25, Lines 734–738):

- *We note that increasing the proliferation rate is scaling the system in time, which enables us to reduce the required simulation time. An alternative to increasing proliferation rate might be to increase the time step, however due to the mechanical parameters of the system this is not feasible in the model as it leads to numerical instability.*

We have also made the scaling more formal as suggested (Page 26, Lines 739–743):

- *The subcellular rate parameters (k_{+1}, k_2, k_{+3}, and k_{-3}) need to be adjusted to account for the size and time scalings. The fit for pH in Equation (22) is already normalised to the thickness of the stratum corneum and therefore this does not need to be changed. The rate parameters are scaled as follows:*

$$\hat{k}_i = \frac{\hat{T}_M}{T_M} k_i,\ \hat{T}_M = \frac{\tau_T}{v_z}, \tag{36}$$

where τ_T is the target corneum thickness, v_z is the expected velocity of the cells given the proliferation rate, $\hat{T}_M$ and T_M are the migration times of the multicellular and single cell systems respectively, and $\hat{k}_i$ and k_i are the rate parameters for the multicellular and single cell systems respectively. We choose $\tau_T = 8$ CD which, given $T_M = 480$ hrs and $v_z \approx 0.05$ $CD.hr^{-1}$ from Miller et al. [35], gives a migration time in the multicellular system of $\hat{T}_M = 160$ hrs. The new rate parameters for the enzyme-substrate reaction ($\hat{k}_{+1}$ and $\hat{k}_2$) are given in Table 1 and the new functions for the enzyme-inhibitor rate parameters are:

$$\hat{k}_{+3} = g_{+3}(\mathrm{pH}) = (15.6pH - 58.5) \times 10^7\ [M^{-1}.hr^{-1}], \tag{37}$$

$$\hat{k}_{-3} = g_{-3}(\mathrm{pH}) = 6.9 \times 10^6\ e^{-3.0pH}\ [hr^{-1}]. \tag{38}$$

29. L636 - The output time step is now a model parameter and should be defined as such.

We have added this parameter to the parameter table (Table 1, Page 22) in the paper as 'Thickness calculation frequency'.

30. L647 - I suggest that the equation that describes how α depends on subcellular variables goes into the main text. This is one of the key assumption in the paper and is somewhat hidden in the Algorithm definition.

We have added the results section as suggested (Page 8, Lines 235–246):

- *The cell's vertical location in the corneum in the multicellular system, i.e. ξ (acting as a proxy for pH), provides the input into the subcellular model. For each cell, at each multicellular timestep, the subcellular model is integrated over the duration of the multicellular model time step to determine the cell's updated CND proportion (s_i). The new proportion of CND (s_i) is returned to the multicellular system and is used to scale the magnitude of the cell's adhesion to its neighbours using the average of the two cell's CND levels:*

$$\alpha_{ij} = \frac{1}{2}(s_i + s_j)\alpha_0 \, , \tag{13}$$

  where α_{ij} is the adhesion coefficient in Equation (11) between cells i and j and α_0 is the normal cell-cell adhesion coefficient. A diagram to illustrate this coupling is shown in Figure 3. As we explain in the Model section, given the increased proliferation rate used in the model, the rate parameters for the multicellular system are scaled in order to reduce the height of the system for computational efficiency (Table 1).

31. Minor typos

i. L29 flow - follow

ii. L32 - missing molecules

iii. the prose is overly colloquial in places - e.g. L 58 - the final pieces of the puzzle

iv. L664 - density - number of cells per unit volume.

These typos have been corrected as follows:

- i/ii. Corrected as recommended.
- iii. We have changed the sentence (Line 63O) to: *The final components of the system shown in Figure 1 are the inhibitor LEKTI and the pH gradient.*
- iv. We have clarified the density (comment iv) by editing the text as follows (Lines 784–786): *Intuitively, one would calculate the surface density (cells per unit area) of the top layer to determine an appropriate value of Δx. However, the simulation output does not show clear layering, and therefore it is difficult to define a surface density.*

32. The authors use phrases such as 'fill period', 'force-based cell removal', 'output time step', 'pull forces' which to the non-initiated read like jargon. It would be better to explain what the principles are and then label them.

We have clarified the definition of these phrases as follows. We have changed to defining a 'filling simulation', rather than a 'fill period', and have clarified the concept in the text (Page 25, Lines 713–718):

- *We are interested in homeostasis of the tissue, rather than tissue development, and so we need to run the model from an established tissue, as in Miller et al. [35]. To generate this established tissue we run a 'filling simulation': a simulation which begins with only the set of stem cells and populates a predefined tissue domain. For the duration of the filling simulation stem cells are restricted to the basal layer and cell removal occurs when cells move above a set (predefined) height.*

For clarity we have moved the definition of force-based cell removal directly after its use (Page 28, Lines 770–772):

- *In order to model desquamation using the degradation of adhesion mechanism, we implement 'force-based cell removal': a 'removal force' is applied to the top cells in the tissue, causing them to separate from the main tissue body.*

We have changed the term 'output time step' to *thickness calculation frequency*, included it in the parameter table (as suggested in comment 29) and clarified its definition in the text (Page 26, Lines 751–753):

- *We update the tissue height every 120 time steps, or once an hour (parameter 'Thickness calculation frequency' in Table 1), due to the high stochasticity in the height of the system, as seen in the Results section.*

We assume the reviewer is referring to the term 'removal force' and the reference to it pulling the cells. We have more clearly linked the definition of the removal force in its first use (in the results section), and clarified it is a vertical force, which would cause a pulling action (Page 8, Lines 247–250):

- *We also need to incorporate a 'removal force': a vertical force that is applied to 'surface cells', located in the top of the tissue, to pull the low adhesion cells upwards off the top of the tissue.*

We have also made it clearer in the definition of the force-based cell removal, as stated in the second dot point of this comment response.

Reviewer #2

This manuscript describes the implementation and results of a multiscale model incorporating components representing i) the dynamics of corneodesmosome adhesion proteins between epidermal cells and ii) a multicellular overlapping sphere model where the adhesion forces between cells are informed by the protein substrate values calculated in part i). The model is used to examine the process of desquamation and its role in homeostasis and also, the effect of LEKTI which can disrupt this process and is associated with skin disease.

General comments

The manuscript is thorough in its description of the two model components and is clearly written and presented. I am not a mathematician, but the equations and methodology underlying the models appears to be rigorous and well justified. The author's have gone to considerable lengths to inform their model parameters from experimental data extracted from the literature.

We thank the reviewer for their review and positive response to the methods and the paper. We have addressed their specific comments on the manuscript below.

Specific comments

1. Although the components of the multiscale model are individually well described, there is no explicit mention of the time steps associated with the subcellular signalling model and the cell-based model. Some discussion of the native time scales associated with each component and the justification of the time splitting technique implemented should be provided (e.g. see Bhattacharya, P., Li, Q., Lacroix, D., Kadirkamanathan, V., & Viceconti, M. (2021). A systematic approach to the scale separation problem in the development of multiscale models. PloS one, 16(5), e0251297 for a recent overview, though earlier related publications do exist).

Following previous multicellular models of the skin the multicellular model component uses a 30 second time step, and we have added this parameter to the parameter table in the paper. The subcellular model uses a finer temporal resolution that is determined using adaptive ODE solvers in the CVODE numerical library with a relative tolerance of 10^{-4}, and absolute tolerance of 10^{-6}. Components are coupled at the common 30 second timestep.

We have made the following changes to the Model section of the paper to clarify this:

- Table 1 on Page 22 now includes the time step for the multicellular model
- Page 26, Lines 744–751: *The coupling and solution of the ODE model follows Algorithm 1. The subcellular degradation model is solved for each cell in the system for each multicellular time step using an adaptive ODE solver in the CVODE numerical library [47]. We set a relative tolerance of 10^{-4} and an absolute tolerance of 10^{-6} for the solver.*

  Coupling between the two scales occurs at the multicellular time step: every 30 seconds, as stated in Table 1. Both the cell location and current tissue height are taken from the multicellular model to determine pH, and consequently the rate parameters.

2. Page 5 "This model uses cell height to determine the local pH. . . ." This is confusing as "cell height" could refer to the physical dimension of a cell, rather than the co-ordinates in the z direction, so would suggest revising for clarity.

We have changed the text to clarify which height we are referring to (Page 6, Line 169):

- *The model uses the cell's vertical location to determine the its local pH.*

3. Page 5 "We assume that the cell velocity is approximately constant through the stratum corneum. . . ." – confusing as it is later stated that cell movement is an emergent outcome of the multicellular model – or is this value assumed just for a preliminary calculation?

The reviewer is correct—cell movement is an emergent outcome of the model. This sentence is referring to an assumption about what is happening *in vivo*, which is an assumption supported by the model results. We have added further text to clarify this (Page 7, Lines 177–183):

- *We investigate the model dynamics for a single cell moving upwards through the corneum by assuming that the cell velocity is approximately constant through the corneum. This assumption is supported by results from our simulations where the variation in a cell's (daily) velocity as it migrates through the corneum is, on average, 5.7% (for the normal tissue described below). If we assume a migration time of 20 days [36] the approximate upwards velocity, normalised by corneum thickness, is* $v_\xi = 0.05 \text{ day}^{-1}$.

4. Page 5 – "In particular, it has been hypothesised that tight junctions at the peripheral of cells..." should read "peripherary?

We thank the reviewer for noticing this and it has been corrected in the text to periphery (Page 7, Lines 195–197, also in Table 3 on Page 23).

5. Figure 3 needs a colour key/legend

The figure (Figure 3, Page 9) has been updated with a legend, and text has been added to the caption to explain the scale:

- *Diagram of the coupling between the multicellular and the subcellular scales. Cells are coloured by their proportion of remaining corneodesmosome. The cell gets its vertical location, normalised by corneum thickness and start height,* (ξ_i) *from the multiscale model, updates the subcellular model parameters and solves for that time step, and then returns the updated proportion of remaining corneodesmosomes (si) to the multicellular model to scale cell-cell adhesion* $(\mathbf{F}^{\mathbf{A}}_{ij})$.

6. Figure 8b – the difference between the two blue and red lines is unclear.

We have added further detail to the caption to explain the difference (now Figure 7, Page 17):

- *(b) Reactant levels of each cell for one time point with 50% normal inhibitor levels* $(i_T = e_T)$ *and 50% abnormal (no) inhibitor levels* $(i_T = 0)$. *The dashed line is the steady state height. The two paths for both* s *(red) and* e *(blue) are the different subcellular system dynamics for cells that have normal inhibitor levels (lower* e *and slower reduction in* s*) compared to cells with no inhibitor (higher* e *and faster reduction in* s*).*
