# Supplementary material for: Multiscale modelling of desquamation in the interfollicular epidermis
Source: PLoS Comput Biol. 2022 Aug 29;18(8):e1010368. doi: 10.1371/journal.pcbi.1010368 (PMC9462764; doi:10.1371/journal.pcbi.1010368)
Supplement: Attachment — Submitted filename: ResponseToReviewers.pdf [file pcbi.1010368.s008.pdf]

Response to reviewers

We apologise to the reviewer for the comments that were not addressed in the previous revisions. We wish to assure them this was completely a technical mistake and we were unaware that we had not transferred all comments into the original response document. We appreciate their understanding and thank them for their time and insightful evaluation of our paper.

In this document we have responded to the remaining comments from the reviewer. The comments are numbered and shown in sans serif font, responses are given in Roman serif font and relevant extracts from the paper are given in *italics* where text that has been added in this revision is shown in green. The line numbers correspond to the revised manuscript (not the tracked changes document).

Reviewer #1

As far as I can tell, you have addressed the first half (roughly) of my comments and feedback, up to and including the point "Figure 8b – the difference between the two blue and red lines is unclear", but not the points that appear after this. I suspect that perhaps there has been an error in accessing the complete set of comments in my review?

We again apologise to the reviewer for this issue and appreciate their understanding on this mistake. It is not clear how this occurred but we want to assure the reviewer that it was an unintentional mistake. We have addressed the remaining comments below.

1. Regarding the results relating to *Distinct proliferative cell niches can be represented by a homogenous population*
Here is seems that the authours have compared simulations where i) cells are given the mean cycling time and ii) a subset of cells (representing stem cells) are randomly assigned a longer cycling time, and concluded that there is no difference. However, my understanding is that "stem cell niches" referred to regions where stem cells were spatially localised/grouped rather than randomly distributed over the cell membrane. In this regard, the authors have not actually explored the effect of spatially localised stem cell niches, and this should be made clear.

The reviewer is correct that, when two populations of proliferative cells (with different cycle times) have been observed, they have been seen in distinct regions of the epidermis (see Sada et al. 2016, `https://doi.org/10.1038/ncb3359`). However, we would not expect this to affect the results of interest, as the thickness results depend predominantly on cell birth rate and not the spatial distribution of the cells. Potentially it would be expected to increase the variation in turnover time and velocities, as cells in the higher proliferative region were 'pushed up' faster than those above slower proliferative regions. However, the average dynamics would not be expected to change. We have added a sentence on this to the paper (page 12, lines 339–344):

- *To implement the two populations, we set 50% of the cells as fast cycling:* $\mathrm{T_C} = \mathrm{T_1}$, *and 50% as slow cycling:* $\mathrm{T_C} = \mathrm{T_1} + \Delta\mathrm{T_C}$. *Allocation of the cycle time was random—we did not account for any clustering of cells of the same proliferative type. Though this clustering has previously been observed in mouse epidermis [7], it would not be expected to have any significant effect on the thickness and average cell dynamics in the model. Simulation results and a detailed analysis are given in Supplementary S1 Text (Section A3)...*

2. Regarding the results relating to *Heterogenous full recovery is more effective than homogenous partial recovery*
Here the authors explore a hypothetical treatment where the the intrinsic value of inhibitor in either all, or a subset of cells is altered, and this property is passed from mother to daughter cells at division. I am not aware how this would relate to a real treatment modality (where I would assume that there is some diffusion of drug from the deeper tissue, in the case of systemic delivery, or the surface in terms of a topical treatment). It would be desirable to see a little more discussion on how this particular computational exploration relates to real treatment for Netherton Syndrome (or possibly other skin disorders).

Currently there is no cure for Netherton Syndrome, the treatment is targeted to the symptoms of the condition rather than the cause. Most treatments address the resulting moisture loss or immune response to the condition. One treatment for Netherton Syndrome that is hypothesised to cause the epidermal cells to compensate for the causal mechanism is phototherapy (patient case studies: Kaminska et al. 2012, `https://doi.org/10.1111/j.1600-0781.2012.00655.x`; Maatouk et al. 2012, `https://doi.org/10.1111/j.1365-2230.2011.04231.x`). This therapy has varying levels of success, though it is not a 'cure', as the treatment is required continually to maintain clinical improvement.

In our paper, we have proposed two outcomes of treatment for the disease: incremental improvement of all cells, or improvement of a proportion of cells. Given that there is little understanding of exactly how these treatments work, we believe these two treatment outcomes cover the two options that could result in clinical improvement (but not complete cure). In reality, one would expect it to be a combination of the two.

We have added text to the paper to expand on this as follows (page 13, lines 419–443):

- *In NS, no treatments currently exist that address the cause of the disease, only the symptoms. One example of a therapy that may affect the system considered here is (narrowband ultraviolet B) phototherapy. In the treatment of psoriasis the same therapy has been observed to induce apoptosis, growth arrest, and possibly allow for DNA repair [39]. In NS patient case studies the treatment has been reported to cause cause 'clinical' or 'marked' improvement for the patients [40,41]. Consequently, it is possible this treatment results in only partial recovery of normal function in the tissue.*

  Partial recovery after treatment, rather than full recovery of normal function, is a scenario that can generally occur in many disease treatments. If we consider a treatment that attempts to restore function at a cellular or subcellular level, it is possible to hypothesise two scenarios which could result in only a partial recovery: partial restoration of normal function in all cells (homogeneous recovery), or restoration of normal function in only a subset of cells (heterogeneous recovery). If we consider the subcellular system described in this paper, the first scenario would be an increase in inhibitor levels across all cells. In the second scenario, only a proportion of cells would recover normal inhibitor levels and remainder would still have reduced inhibitor levels. It is also probable that the response would be a combination of the two scenarios: different levels of improvement in different subsets of cells.

  In this section we compare the two limiting scenarios: heterogeneous and homogeneous recovery, for a hypothetical treatment that restores some level of inhibitor to cells in the system. The efficacy of homogeneous recovery can be seen above in Figure 5b where results show a linear relationship between the inhibitor concentration in the cells and the tissue thickness. For heterogeneous recovery, we assume that the level of inhibitor in any cell is inherited from its parent stem cell and assign a level of inhibitor to each stem cell...

3. It is mentioned that only 10 simulations per case were carried out. Was this due to prohibitive computational resources requirements/run times? It would be interesting to see more information on this, even if

only in Supplementary material.

The reviewer is correct that it was due to the available computational resources and the requirements of the simulations. Each simulation takes between 42 hours and 58 hours to run and a total of 150 simulations were required to get 10 simulations per case (the total computational time for all 150 simulations was 327 days). The variation is due to the different tissue heights (thicker tissue has longer computational times). We had capacity on the available server for 10 simulations at once, so around 1 month to get all the results. The expectation was that to provide any decent scale-up would require significantly more resources but would not result in any difference in conclusions (simply a refinement of the metrics). We have added a sentence on this in the Model section of the paper:

- (page 26, lines 736–738): *Due to stochasticity in the proliferation cycle, 10 realisations are run for each simulation setup. This number is chosen as a balance between reliability of the results and the available computational resources (individual simulation times range from 42 to 58 hours depending on resulting tissue thickness).*

4. There is no parameter sensitivity analysis provided on which to judge the robustness of the results. This should at least be discussed as a limitation to the work.

A coarse (orders of magnitude) parameter sweep investigation into the sensitivity of the subcellular system to the reactant concentrations is given in the Supplementary Material (Section A2). This analysis also provides some likely hypotheses as to the sensitivity of changing the rate parameters of the equations (as the effective rate parameter is the product of the concentrations and rate parameters; see Equations 16–21 in the main paper). The reviewer is right that it would be informative to run a formal sensitivity analysis on both these concentrations and the rate parameters using the full multiscale model, however this will be a significant computational undertaking and so we consider it a current limitation and are interested in performing such an analysis as future work. We have added a sentence to the limitations and future work on this (Page 19, lines 541–544):

- *Consequently, as potential improved testing methodologies, or more comprehensive data, become available it will be possible to improve this model. We are also interested in performing a parameter sensitivity analysis on the full system, for the subcellular parameters, extending the work presented in Supplementary S1 Text (Section A2), to better understand the sensitivity of the model results to each of the rate parameters and reactant concentrations..*

5. Model Description
In the subcellular model, it does not appear that new protein production, or degradation is accounted for. This may be justified by the timescales, but given that they are not explicitly stated, it is difficult to tell.

We would not expect new protein production to occur in the corneum as the cells in the corneum are no longer metabolically functioning (and have no nucleus). The proteins are secreted by the cells at the interface between the granular layer and the corneum. However, the reviewer is right that it is possible that enzyme degradation occurs in the corneum. We have added a sentence to the paper (page 20, lines 583–585):

- *... All rate parameters, k_{+1}, k_{-1}, k_2, k_{+3}, and k_{-3}, are initially assumed to be functions of local pH. We also assume there is no degradation of the inhibitor or enzyme over the time scale of the cell's migration time through the corneum (approximately 20 days in the epidermis [36]).*

6. Page 19 paragraph 1 - typo – "...which is discussed in the The multiscale model section."

This has been changed to (page 21, line 599): *...which is discussed in the Multiscale model section.*

7. Another assumption is that extracellular space between cells does not vary with z position within the epidermis. This is unlikely to be the case, so should be mentioned as a simplification.

We added a sentence on this simplification in the paper as follows (page 22, line 639–641):

- *This was done using an approximate molecular weight of KLK5, the water content of the corneum, and the volume ratio of cell to extracellular space in the corneum. As a simplification, due to limited data, we assume that the volume of the cell and the extracellular space is constant throughout the corneum. Values for all of these parameters are given in Table 2.*

8. Page 21 paragraph 1 typo – "..assume that any enzyme that is not complex with the inhibitor...*

We have fixed the typo (page 23, line 661): *...assume that any enzyme that is not in complex with the inhibitor...*

9. Page 22 – equation 19 – the formulation of the force terms should be justified/referenced.

This was addressed in the previous revisions. The following details were added (page 24, line 693):

- *The intercellular adhesion and repulsion forces are based on functions from Atwell et al. [49] and Li et al. [34] respectively...*
